# Spider silk proteome provides insight into the structural characterization of *Nephila clavipes* flagelliform spidroin

**DOI:** 10.1038/s41598-018-33068-9

**Published:** 2018-10-02

**Authors:** José Roberto Aparecido dos Santos-Pinto, Helen Andrade Arcuri, Franciele Grego Esteves, Mario Sergio Palma, Gert Lubec

**Affiliations:** 1 Center of the Study of Social Insects, Department of Biology, Institute of Biosciences of Rio Claro, São Paulo State University, Rio Claro, SP 13500 Brazil; 2 Paracelsus Medical University, A 5020 Salzburg, Austria

## Abstract

The capture spiral of web from *N. clavipes* spider consists of a single type of spidroin - the flagelliform silk protein, a natural material representing a combination of strength and high elasticity. Flagelliform spider silk is the most extensible silk fibre produced by orb weaver spiders and the structure of this remarkable material is still largely unknown. In the present study we used a proteomic approach to elucidate the complete sequence and the post-translational modifications of flagelliform silk proteins. The long sequence of flagelliform silk protein presents 45 hydroxylated proline residues, which may contribute to explain the mechanoelastic property of these fibres, since they are located in the GPGGX motif. The 3D-structure of the protein was modelled considering the three domains together, i.e., the N- and C-terminal non-repetitive domains, and the central repetitive domain. In the resulting molecular model there is a predominance of random structures in the solid fibres of the silk protein. The N-terminal domain is composed of three α-helices and the C-terminal domain is composed of one small helical section. Proteomic data reported herein may be relevant for the development of novel approaches for the synthetic or recombinant production of novel silk-based spider polymers.

## Introduction

Produced by flagelliform gland, the flagelliform silk protein (FSP) forms the silk fibres of the orbital web’s capturing spiral. This part of the silk is highly elastic and in conjunction with the silk properties of the frame and the ray of the orbital web, dissipates the energy of prey impacting on the web. The enormous resistance of these fibres is important for the capture and arrest of prey that is sometimes larger than spiders themselves^1,2^.

The FSP has the sequence repeat GPGGX, GGX and a spacer sequence composed of residues of charged amino acids and is highly conserved. According to Becker *et al*.^3^ the crosslinks between silk proteins may come from spacer sequences in the flagelliform silk protein. Adrianos *et al*.^4^ described a series of mechanical tests of the recombinant FSP, in which the GGX motif contributes to extensibility, while the spacer motif contributes to the strength of the recombinant fibres - according to the authors likely a primary contributor of strength, with the GGX motif supplying mobility to the protein network of native *N. clavipes* flagelliform silk fibres.

Up to now no complete structural model was determined/proposed for FSP; since there is a lack of data regarding to the real silk protein structures in their native state. Unlike the other protein constituents of the silk, the FSP does not undergo conformational changes during the spinning process^2^. There is limited information about the structures constituted by the GPGGX and GGX repeated sequences in the FSP; however, some studies describe these regions as amorphous with no crystalline structures in the fibres, and conformations without preferential orientation^1,5,6^; the abundance of proline residues regularly distributed throughout the repeat GPGGX sequence, prevents the formation of crystalline β-sheet structures in the fibre^7,8^. In contrast, in other studies have been proposed, for the GPGGX motif, the formation of a 3_10_-helical structure or β-spiral structures; for this last one is suggested that can act as molecular nanosprings providing elasticity to the fibres^6^. In addition, studies by Perea *et al*.^9^ demonstrated the presence of polyglycine II nanocrystals in *Argiope trifasciata* flagelliform silk. According to the authors, the polyglycine dominant motifs GGX and GPGGX can contribute to increase toughness and achieve the ability of flagelliform silk to supercontract; this is possible due the conformational changes induced by the stretching step that leads the protein to a molecular reorganization of spatially close chains. As we can note, despite of these studies regarding to the FSP structure, still it is not clear the presence or absence of ordered regions in their fibres; it is necessary more chemical information and structural data for a better comprehension of FSP mechanoelastic properties.

It is known that spider silk can be used for many applications due of mechanical and physicochemical properties of spidroins; thus, the spider silk has been produced by recombinant DNA technology^10–12^. However, there exists limited information about post-translational modifications (PTMs), for example. So far, all work on mechanical properties of the silk fibres has been carried out with no consideration of the presence of PTMs in the spidroin sequences. Modifications such as phosphorylation^13–16^ and glycosylation^17,18^ have been reported in silk from spiders. Available studies have used mass spectrometry with different experimental approaches for the identification and location of PTMs in most diverse biological samples^14–16,19–23^. In the present study, an experimental approach was adopted combining 2-DE gels with multiple proteolytic in-gel digestion, followed by mass spectrometry (MS^n^) analysis using two different fragmentation methods, collision-induced dissociation (CID) and electron-transfer dissociation (ETD) for the characterization of FSP. Phosphorylation at tyrosine residues, for example, is considered relatively stable during MS^n^ fragmentation, whereas at serine and threonine residues it has a moderate stability. Nitrotyrosine is considered to be unstable to moderately stable, and hydroxyproline is considered stable^24–26^. Therefore, all these modifications are detectable also by CID as observed in the current study.

**Figure 1:**
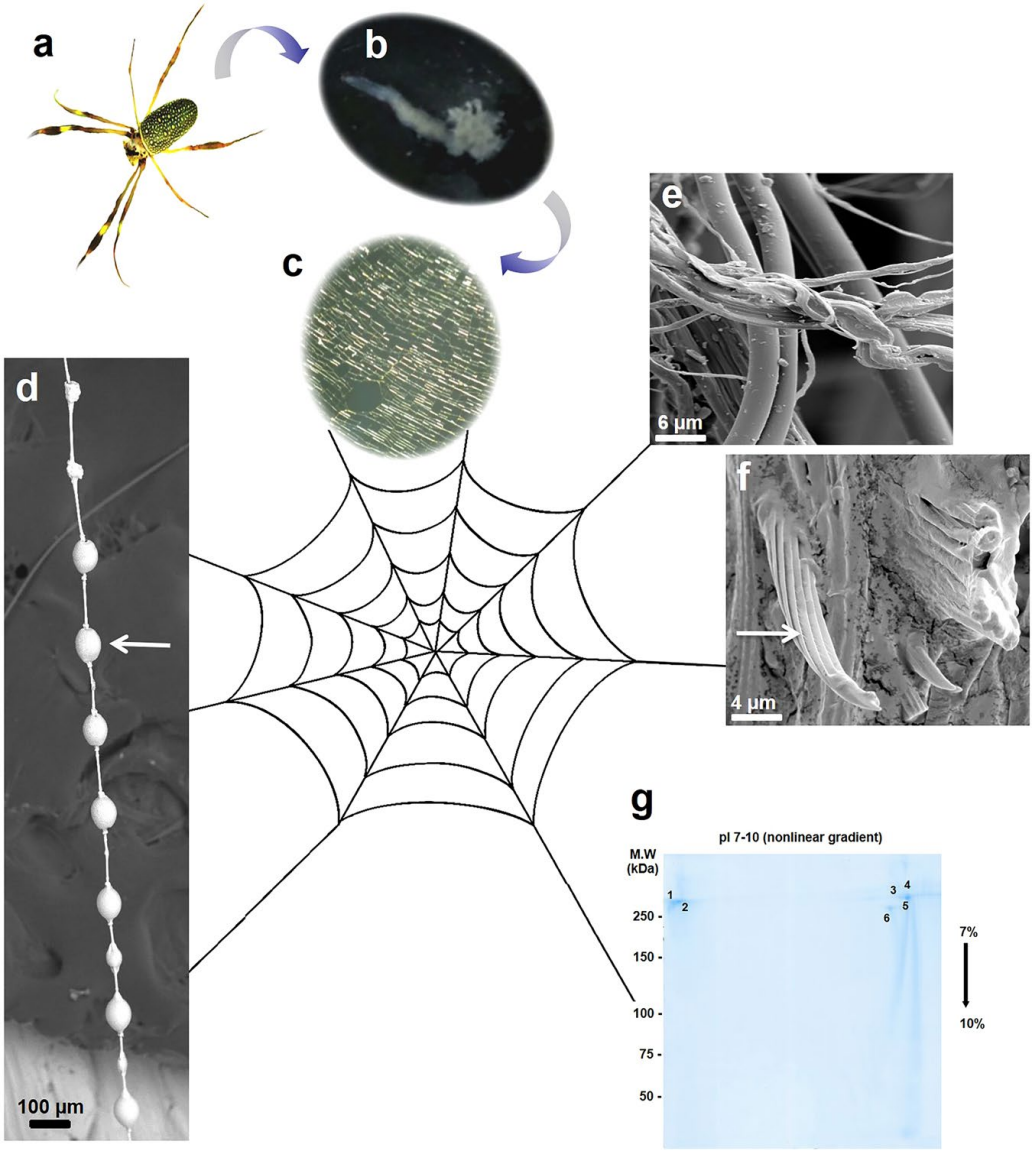
Scanning electron microscopy analysis of the web silk produced by (**a)** *N. clavipes spider*; (**b)** flagelliform gland of the *N. clavipes* spider; (**c)** in detail the yellowish colored fibres, secreted by the flagelliform gland, that form the capture spiral of the orbital web; (**d)** Ultrastructure of the flagelliform silk (spiral) highlighting the droplets (white arrow) on the fibre. (**e**,**f**) Solubilization in lithium thiocyanate highlighting the layer structures (white arrow) of silk. (**g**) Representative 2-DE profile of the flagelliform silk stained with *Coomassie Colloidal Blue* (From original image demonstrated in Supplementary Information - Figure S7).

Considering that the sequence as well as the identification of the post-translational modifications (PTMs) of FSP produced by orb weaver spiders are not completely known, the web silk protein produced by *N. clavipes* spider (Fig. 1a,b) was submitted to proteomic analysis for its structural characterization. The complete sequence and the PTMs assigned in the present study were used to simulate the 3-D structure of the FSP from the capture spiral (Fig. 1c) under natural conditions.

## Results

### Morphological and structural characterization of silk fibres

Scanning electron microscopy (SEM) analysis of the web silk protein demonstrated the fibres ultrastructure at differentiated capture spiral positions (Fig. 1d–f) and showed that these fibres comprise compacted forms of silk layers. Figure 1d shows an SEM image of several droplets on the capture spiral fibre. As the flagelliform silk fibres are expelled by the flagelliform gland (Fig. 1b) a highly viscous coating is simultaneously deposited by the aggregate gland on these fibres, forming droplets^27,28^. The solubilization of silk fibres in lithium thiocyanate was a key step for the dissolution of the silk proteins prior to performing the 2-DE electrophoresis, used as strategy for proteomic analysis of this very large protein.

### Flagelliform silk protein sequencing

The electrophoretic profile of the solubilized web silk (Fig. 1g) revealed six spots of spidroins (labelled 1 to 6) with apparent molecular weights higher than 250 kDa. The proteins in these spots were sequenced using mass spectrometry and identified in the gel as follows: FSP (spots 1 and 2) (GenBank ID accession numbers O44358 and O44359); spidroin-1A (spot 6) (GenBank ID accession numbers B5SYS5 and P19837); and spidroin-1B (spots 3, 4 and 5) (GenBank ID accession numbers B5SYS6 and P19837). The spidroins-1A and -1B, produced in the major ampullate glands, are present only in the frame and radial threads of the web; while the FSP, produced by the flagelliform gland, is present in the capture spiral. The analyses were carried out with the FSP identified in the spots 1 and 2; all the sequence obtained are shown in Tables S1 and S2. The sequence assignments of spidroins-1A and -1B are not presented in this study, since they were reported in a previous study^15^.

A gel-based mass spectrometry strategy with collision-induced dissociation (CID) fragmentation method was applied to sequence flagelliform silk protein (including the N- and C-terminal non-repetitive domains and the central repetitive domain) and to check the presence/position of PTMs within the FSP sequence. The spidroins (spots 1 and 2) were cleaved using different proteases and subjected to tandem mass spectrometry analysis. This allowed the identification and sequence assignment of 292 proteolytic peptides related to the FSP sequence: 5 tryptic peptides, 164 chymotryptic peptides, 29 proteolytic peptides were detected after digestion with Glu-C/V8 protease, 9 proteolytic peptides from digestion with subtilisin protease and 75 proteolytic peptides from digestion with proteinase 10. All peptide fragments (Tables S1 and S2) were identified by only two Accession Numbers: O44358 (a large fragment containing the N-terminal and central domains sequences) and O44359 (a large fragment containing the central and C-terminal domains sequences). The Table S3 shows the list of the 185 non-redundant proteolytic peptides in the chromatograms generated by the digestion of flagelliform silk protein with trypsin, chymotrypsin, Glu-C/V8 protease, proteinase 10 and subtilisin, presented according to the domain region to which they belong to (non-repetitive N-terminal, central repetitive, and non-repetitive C-terminal domains) along the sequence of *N. clavipes*.

The sequences of these peptides were aligned with the sequences of three large FSP fragments, corresponding to different domains of *N. clavipes* FSPs deposited in UniProtKB (http://www.uniprot.org/help/uniprotkb) (Fig. S1): (i) accession number O44358 - fragment corresponding to the non-repetitive N-terminal domain (NR-NTD); (ii) accession number Q9NHW4 - fragment corresponding to the most of central repetitive domain (CRD); and (iii) accession number O44359 - fragment corresponding to the central repetitive domain (CRD), as well the non-repetitive C-terminal domain (NR-CTD). The overall alignments revealed an identity of 89% of the experimental sequence of *N. clavipes* FSP determined in the present investigation, in relation to the overall sequence of the three domains deposited in UniProtKB.

### Semi-quantitative analysis of peptide sequences

There are some peptide sequences that occurred many times all over *N. clavipes* FSP, while other peptides occurred only once; the Table S3 shows the exact origin of each peptide along FSP sequence. Thus, in order to verify if the relative abundance of each one of the 185 non-redundant proteolytic peptides were proportional to their occurrence in the FSP sequence, the peptides were semi-quantified using the spectral counting strategy. Technically, the absolute quantification of these peptides, is difficult to be performed; for that it would be necessary to isolate each peptide for individual quantification; thus, it was used a semi-quantitative method based on spectral counting strategy. It is important to emphasize that the amount of each peptide in the chromatogram is reflecting its occurrence along the FSP sequence; and it makes the *N. clavipes* FSP sequencing highly probable. Table S3 shows the integer value of the normalized spectral counting during mass spectrometry analysis of each peptide, which is reflecting directly the number of peptides occurrence in *N. clavipes* FSP sequence. This table also shows the *m/z* values of the respective molecular ions, charge state, and the position of each peptide along the sequence. Thus, the FSP sequencing results seem to be trustworthy, and apparently, the proteolytic digestion used for generating the peptides (tools of sequencing) reached the fullness. As a consequence of FSP sequencing, it was possible to identify, between the N- and C-terminal domains, three modules of the central repetitive domain in tandem, (Fig. S1). A careful observation of alignments of all experimental sequences and those of three large FSP fragments revealed that the most of these sequences are complementary to each other. The calculated MW for *N. clavipes* FSP sequence as shown in figure S1, is 195 kDa; considering that the apparent MW values observed for protein spots in 2-DE gel profile is about 266 kDa (Fig. 1g), it is possible to speculate that the FSP present in electrophoresis sample presented four CRD modules, while that analysed by a proteomic approach presented three CRD modules.

### Post-translational modifications assignment

High-sequence coverage of FSP alllowed the identification of a series of post-translational modifications (PTMs) and amino acid substitutions. The MASCOT protein engine search and Modiro^**®**^ were used to analyse all generated spectral data allowing the assignment of PTMs and amino acid substitutions such as S244Y, V396L, A444V, E503A, S512P, Y513D, G676R, S677Y, V819L, S979Y, V1131L, A1179V, E1238A, S1247P, Y1248D, G1411R, S1412Y, V1554L, S1714Y, V1866L, A1914V, E1973A, S1982P, Y1983D, G2146R, S2147Y, V2289L, G2341S, Y2346N and N2411D in the sequence of *N. clavipes* FSP (Tables S2 and S3). The detection of amino acid substitutions may be due to single nucleotide polymorphism, mutations or just mean the change in the amino acid composition of FSP in response to prey variation. Some studies show that spider silk proteins expression can be altered due to the interaction among the location, diet and type of prey^29,30^ and furthermore that these substitutions may change protein conformation and possibly even the mechanical properties of silk fibres. Tables, S1 and S2, are showing detailed proteomic data including all of PTMs assignments. During sample preparation or analytical procedures, some modifications such as oxidation, deamidation and methylation might have occurred due to various artefacts However, 45 sites of hydroxyproline, 9 sites of phosphotyrosine and 3 sites of nitrotyrosine were the major PTMs assigned to the FSP sequence (Table 1).

**Table 1:** Mapping of the hydroxyproline, phosphotyrosine and nitrotyrosine sites observed on *N. clavipes* flagelliform silk protein. All the positions of these modifications are demonstrated in Fig. 4, FSP sequence.

PTMs	Enzyme	Fragmentation method	Comments
**Hydroxyproline**			
P136, P871, P1606	Chymotrypsin	CID	Table S2 (*spot* 2)
P146, P881, P1616	Chymotrypsin	CID	Table S2 (*spot* 2)
P156, P891, P1626	Chymotrypsin	CID	Table S2 (*spot* 2)
P166, P901, P1636	Chymotrypsin	CID	Table S2 (*spot* 2)
P176, P911, P1646	Chymotrypsin	CID	Table S2 (*spot* 2)
P191, P926, P1661	Chymotrypsin	CID	Table S2 (*spot* 2)
P524, P1259, P1994	Chymotrypsin	CID	Table S2 (*spot* 2)
P559, P1294, P2029	Chymotrypsin	CID	Table S2 (*spot* 2)
P579, P1314, P2049	Chymotrypsin	CID	Table S2 (*spot* 2)
P589, P1324, P2059	Chymotrypsin	CID	Table S2 (*spot* 2)
P599, P1334, P2069	Chymotrypsin	CID	Table S2 (*spot* 2)
P609, P1344, P2079	Chymotrypsin	CID	Table S2 (*spot* 2)
P629, P1364, P2099	Chymotrypsin	CID	Table S2 (*spot* 2)
P639, P1374, P2109	Chymotrypsin	CID	Table S2 (*spot* 2)
P709, P1444, P2179	Chymotrypsin	CID	Table S2 (*spot* 2)
**Phosphotyrosine**			
Y489, Y1224, Y1959	Chymotrypsin	CID	Table S1 (*spot* 1)
Y498, Y1233, Y1968	Chymotrypsin	CID	Table S1 (*spot* 2)
**Nitrotyrosine**			
Y445, Y1180, Y1915	Chymotrypsin	CID	Table S2 (*spot* 1)

**Figure 2:**
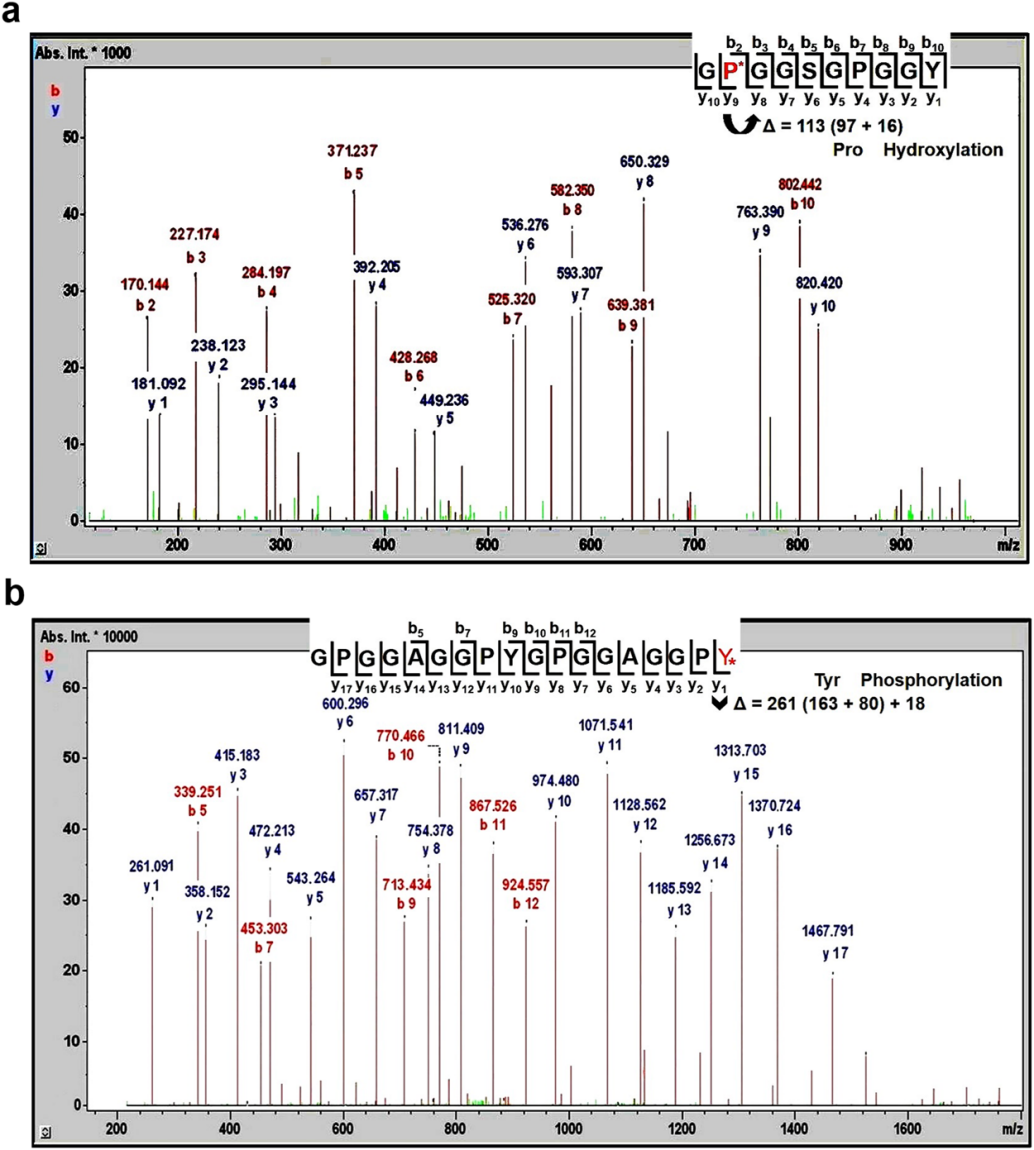
Representative mass spectra of *N. clavipes* flagelliform silk protein. (**a**) CID spectrum of the chymotryptic peptide GP^*^GGSGPGGY (135–144, 145–154, 155–164, 165–174, 175–184, 190–199, 558–567, 578–587, 588–597, 598–607, 608–617, 628–637, 638–647, 708–617, 870–879, 880–889, 890–899, 900–909, 910–919, 925–934, 1293–1302, 1313–1322, 1323–1332, 1333–1342, 1343–1352, 1363–1372, 1373–1382, 1443–1452, 1605–1614, 1615–1624, 1625–1634, 1635–1644, 1645–1654, 1660–1669, 2028–2037, 2048–2057, 2058–2067, 2068–2077, 2078–2087, 2098–2107, 2108–2117, 2178–2187), which was acquired by selecting the m/z 411.210 [M + 2 H]^2+^ as a precursor ion and showing the hydroxyproline sites at (P136, P146, P156, P166, P176, P191, P559, P579, P589, P599, P609, P629, P639, P709, P871, P881, P891, P901, P911, P926, P1444, P1294, P1314, P1324, P1334, P1344, P1364, P1374, P1606, P1616, P1626, P1636, P1646, P1661, P2029, P2049, P2059, P2069, P2079, P2099, P2109 and P2179). (**b**) CID spectrum of the chymotryptic peptide GPGGAGGPYGPGGAGGPY* (481–498, 1216–1233, 1951–1968), which was acquired by selecting the m/z 763.411 [M + 2 H]^2+^ as a precursor ion and showing the phosphorylation sites at Y498, Y1233 and Y1968.

The Fig. 2a,b shows two representative spectra that present the assignment of hydroxyproline and phosphotyrosyne sites. Thus, Fig. 2a shows the CID spectrum of the chymotryptic peptide GP^*^GGSGPGGY (occurring at the positions 135–144, 145–154, 155–164, 165–174, 175–184, 190–199, 558–567, 578–587, 588–597, 598–607, 608–617, 628–637, 638–647, 708-617, 870–879, 880–889, 890–899, 900–909, 910–919, 925–934, 1293–1302, 1313–1322, 1323–1332, 1333–1342, 1343–1352, 1363–1372, 1373–1382, 1443–1452, 1605–1614, 1615–1624, 1625–1634, 1635–1644, 1645–1654, 1660–1669, 2028–2037, 2048–2057, 2058–2067, 2068–2077, 2078–2087, 2098–2107, 2108–2117, 2178–2187) used to assign the hydroxyproline sites on *P136, *P146, *P156, *P166, *P176, *P191, *P559, *P579, *P589, *P599, *P609, *P629, *P639, *P709, *P871, *P881, *P891, *P901, *P911, *P926, *P1444, *P1294, *P1314, *P1324, *P1334, *P1344, *P1364, *P1374, *P1606, *P1616, *P1626, *P1636, *P1646, *P1661, *P2029, *P2049, *P2059, *P2069, *P2079, *P2099, *P2109 and *P2179) (Table S3), while Fig. 2b shows the CID spectrum of the chymotryptic peptide GPGGAGGPYGPGGAGGPY* (occurring at the positions 481–498, 1216–1233, 1951–1968) (Table S3), used to assign the phosphotyrosine sites on *Y498, *Y1233 and *Y1968. Since there is no sufficient space to present all spectra used for each PTM site assignment, others representative spectra are also shown in figures S2–5.it is important to emphasize that in the current study we are using a numbering sequence based on the protein fragments used for alignment of *N. clavipes* FSP sequence (Table S3), while in protein databases the numbering standard refers to each partial sequence.

**Figure 3:**
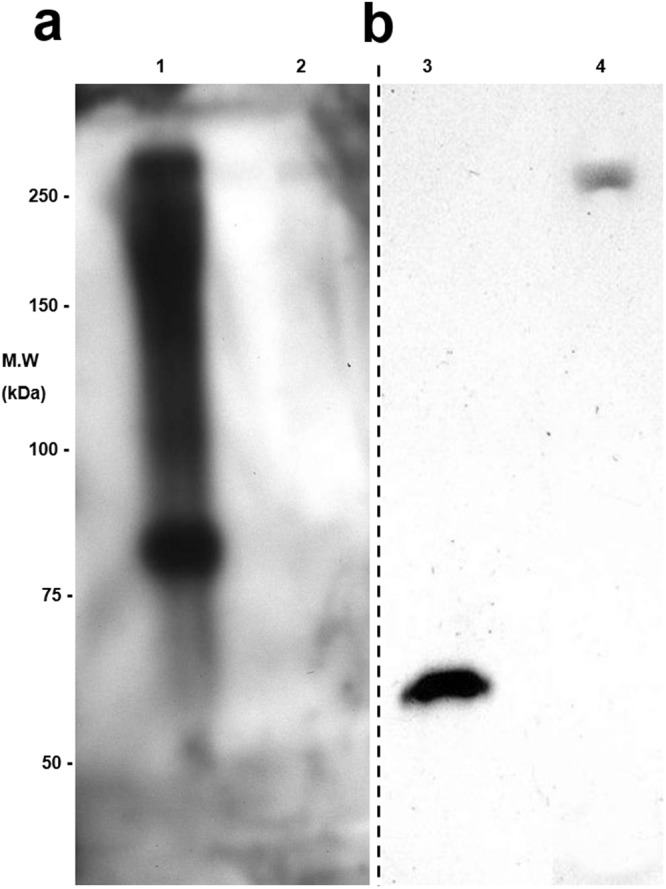
Proteomic analysis of *N. clavipes* web silk. (**a**) Western blotting showing phosphotyrosine immunoreactivity (lane 1). Lane 2 shows no immunoreactivity after phosphatase treatment. (**b**) Western blotting showing nitrotyrosine immunoreactivity (lane 4). Lane 3 shows control sample Nitro-BSA (Nitrated bovine serine albumin, Sigma) (From original images demonstrated in Supplementary Information - Figures S8 and S9).

**Figure 4:**
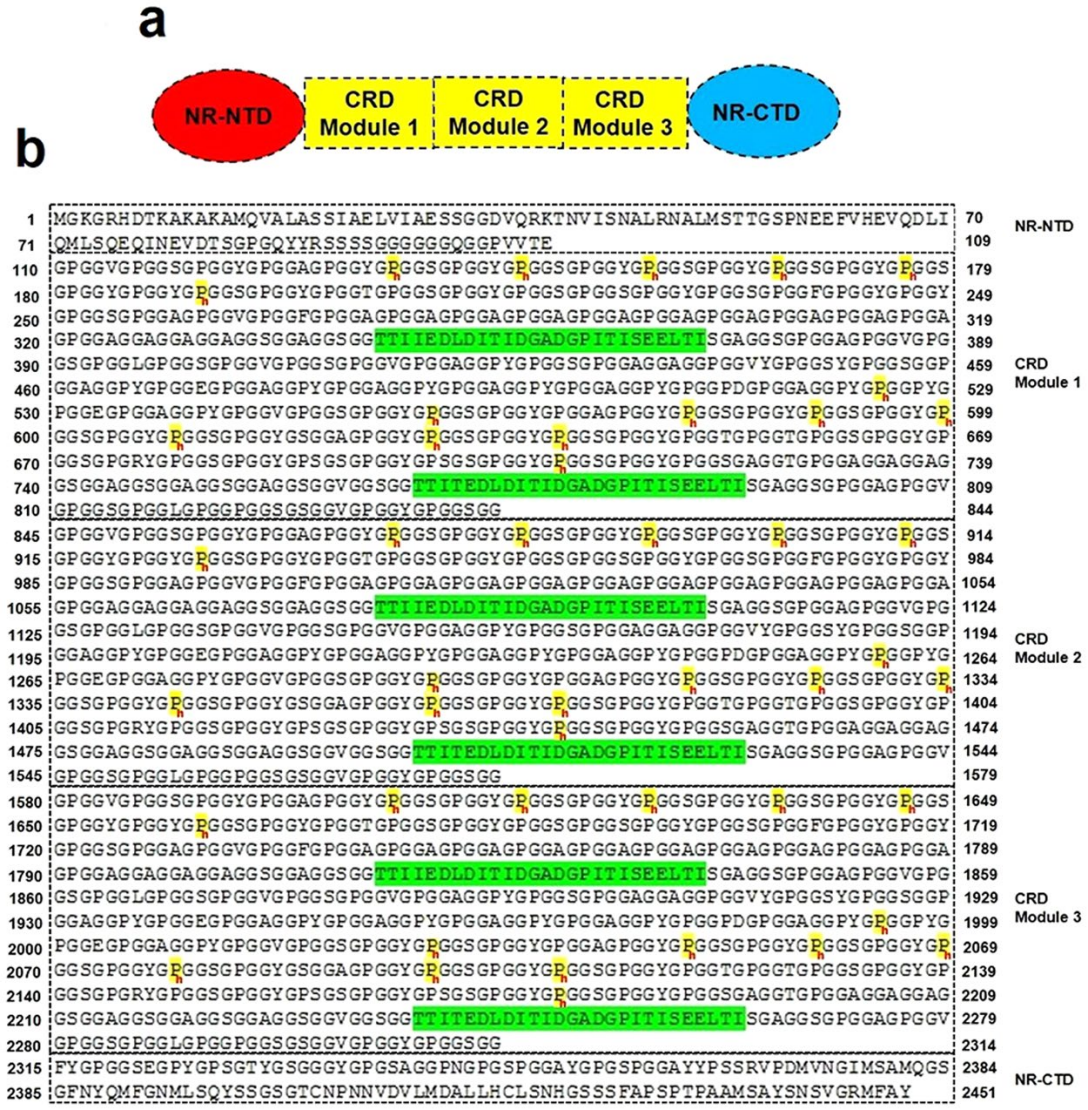
Representative sequence of *N. clavipes* FSP, highlighting the Non-Repetitive N-terminal Domain (NR-NTD), the three modules of the Central Repetitive Domain (CRD), and the Non-Repetitive C-Terminal Domain (NR-CTD). The hydroxyproline sites are assigned in yellow and marked with red “h” letter; the “spacer” domain is assigned in green.

Phosphotyrosine sites were also detected and confirmed by immunoblotting, using IgG anti-phosphotyrosine for staining. Figure 3a is showing an intense staining observed for the protein without any treatment, in its turn the protein treatment with alkaline phosphatase removes the staining totally; as well as nitrotyrosine sites were also detected and confirmed by immunoblotting, using IgG anti-nitrotyrosine for staining. In this case, Nitro-BSA (Nitrated bovine serum albumin MW 68 kDa, Sigma) was used as positive control (Fig. 3b). Figure 4a,b shows the *N. clavipes* FSP sequence, indicating the N- and C-terminal domains as well as three modules of the repetitive central domain with the assignment of hydroxyproline positions for the FSP.

### 3D structure of the flagelliform silk protein

**Figure 5:**
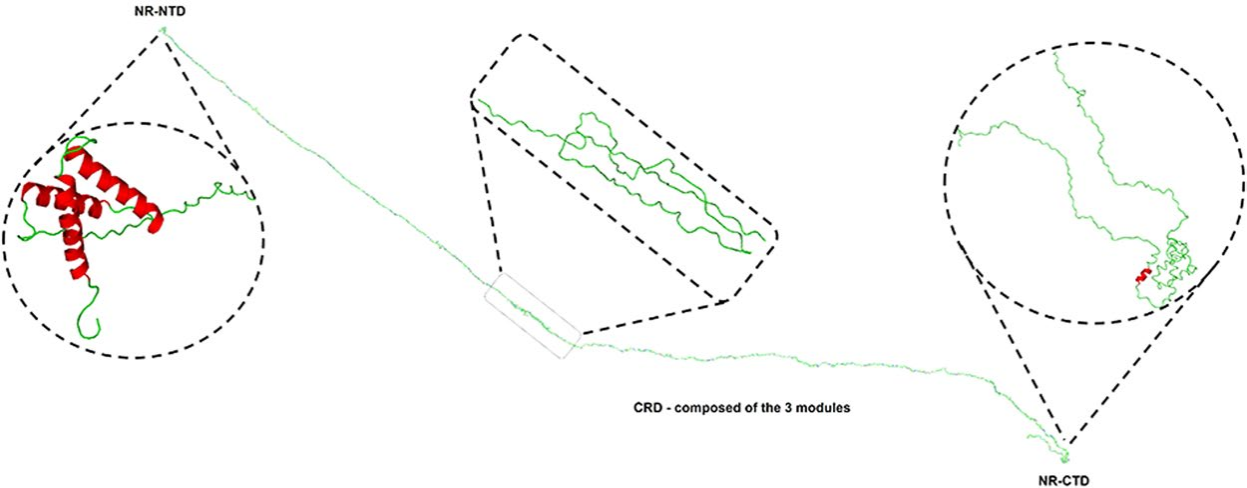
Representative three-dimensional molecular model of FSP, constituent of the silk fibres from *N. clavipes* spider. In details the N- and C-terminal non-repetitive domains, and the central repetitive domain, composed of the three modules of the primary sequence, as shown in Fig. 4.

Given the results provided by the identification and extensive sequencing of FSP, molecular modelling and molecular dynamics simulations for this protein were performed to obtain structural information to support the understanding of mechanical and physicochemical properties of silk fibres from *N. clavipes*. FSP was modelled using the following proteins as templates: PDB ID: 2LPJ (major ampullate spidroin 1 N-terminal domain)^31^, 3HQV and 3HR2 (type I collagen)^32^, and 2MFZ (C-terminal domain of minor ampullate spidroin)^33^. Considering that FSP may get direct contact with large amounts of water from air humidity and/or rain under natural conditions, the protein was submitted to molecular dynamics (MD) simulations in presence of water. Thus, our simulations results are more adequate for FSP in solution, than in the fibres, where could occur interaction among the proteins. For simulation of protein-protein interaction, it would be necessary to perform the MD using a specific force field for this purpose, in a system with very high computation power to complete all analyses. The MD simulations were run for 10 ns; after this time, all model validation parameters (protein backbone RMSD, radius of gyration, potential energy analysis, and number of hydrogen bonds) revealed that the molecular model was reliable (Fig. S6a–d). The secondary structure analysis of *N. clavipes* FSP was performed using the JNET *Secondary Structure Prediction* program and PROCHECK, which indicated that FSP exhibits a heterogeneous, disordered and randomly oriented structural conformation. There is predominance of random structures in the solid fibres of the silk; the N-terminal domain is composed of 3 α-helices and the C-terminal domain is composed of 1 small helical section. The proposed 3D structure of the molecular model of the FSP was generated from the PyMol program^34^ and is shown in Fig. 5.

## Discussion

The sequence of *N. clavipes* FSP has been analyzed by mRNA and cDNA analysis^35,36^ and it is known that spidroins in general contain a large repetitive core sequence, which is flanked by non-repetitive N- and C-terminal domains^37^. Considering this, the aim of the present study was to characterize the FSP structure (including the non-repetitive N- and C-terminal domains and the central core) by a proteomic approach.

The protein databases GenBank (http://www.ncbi.nlm.nih.gov/genbank/submit) and Uniprot (http://www.uniprot.org/help/uniprotkb) contain many entries for different spidroin fragments that were virtually translated from DNA sequences and deposited in databases. Therefore, our strategy for sequencing FSP was to cover as much as possible the sequence, based on the proteolytic fragments from spots 1 and 2 (Fig. 1g); and overlapping the peptide sequences using the sequence informations deposited in GenBank and UniProt to achieve the highest possible coverage.

Our sequencing results demonstrated that the MW calculated for the sequence of *N*. *clavipes* FSP is 195 kDa; studies have shown that the spidroins of silk fibres have MW around 250–300 kDa after being secreted by the spinning duct forming the solid fibres^38,39^. The apparent MW of 266 kDa obtained in the denaturing gels is consistent with *N*. *clavipes* FSP presenting NR-NTD and NR-CTD flanking four modules of CRDs, while the sequence shown in Fig. 4 is showing the presence of three modules of CRDs. The number of CRD modules was previously reported to change from three to six for the spidroins^15,16^, which may explain the results reported above.

Another aspect that must be emphasized is that all the PTMs detected in the present investigation occurred in the CRDs (Fig. 4 and Table S2). Denaturing gels revealed by phosphotyrosine-immunoreactivity (Fig. 3) demonstrated a band around 85 kDa, which was not detected in a 2-DE (possibly because it is present in minimal quantities in the silk fibres). This MW value seems to be consistent with an FSP fragment corresponding either to NR-NTD or NR-CTD associated to one module of CRD. In the present investigation, a total of 9 phosphotyrosine sites were observed on FSP that were initially assigned by using CID fragmentations in mass spectrometric analysis. The assignments of phosphotyrosine residues were confirmed in *N. clavipes* FSP by immunoblotting, in absence and presence of treatment with alkaline phosphatase (Fig. 3a). It is important to note that, some proteomic studies have demonstrated the presence of phosphorylation on some known silk proteins including the fibroin from *Bombyx mori* (i.e. the heavy and light chain of fibroins, and the P25 protein)^19,40,41^ and the spidroin-1 protein from *N. clavipes, N. madagascariensis* and *N. edulis* spiders^14^. In our own studies, published recently^15,16^, in *N. clavipes* spider silk a total of 15 and 16 phosphorylation sites were identified on spidroin-1A and −1B, respectively; and a total of 36 phosphorylation sites on spidroin-2. So far, it is not clear the biological significance of many phosphorylation sites observed on the spider silk proteins. In spite that, based on literature data suggests that such modifications might be related to the structural conformation domains which are responsible for the mechanoelastic properties of spider silk fibres.

Nitrotyrosination sites were also observed on FSP which was confirmed by immunoblotting (Fig. 3b). The tyrosine oxidations were reported to form oxidation-mediated cross-links from two tyrosine radicals^42^. The functional relevance of nitrotyrosinations observed on FSP in the present study remains unclear, but previous work revealed the effects of tyrosine nitration on mechanoelastic properties and on protein-protein interactions^21,43^.

Hydroxyproline was the major PTM reported in the present study, with a total of 45 sites assigned to the FSP sequence. This modification consists of the addition of hydroxyl groups to the proline residues catalyzed by prolyl hydroxylase (PHD), warranting protein stability^44^. The *N. clavipes* web-silk is known to be highly elastic and the observation of a large amount of hydroxylated proline residues could potentially explain some elastic properties presented by the web fibres, just as it occurs in collagen found in the mussel *Mytilus edulis*^45,46^. Hydroxyproline is an essential amino acid residue present in collagen and studies indicate the presence of structural domains in the collagen of *M. edulis*, similar to the domains observed in spider silk; these domains may increase strength and extensibility of collagen^45–47^. Sutherland *et al*.^48^ have also demonstrated the presence of the structural motif of collagen in the insect silk willow sawfly, *Nematus oligospilus* (Hymenoptera) and has been characterized as collagen silk proteins.

In the case of FSP hydroxyprolines are located in the GPGGX motif, probably representing the formation of β-spiral structures that can act as molecular nanosprings providing elasticity to the fibres^5^. Previous work has shown that FSP does not undergo conformational changes during the spinning process, the vast abundance of proline residues regularly distributed throughout the repeat GPGGX sequence, prevents the formation of crystalline β-sheet structures in the fibre^7,8^. Previous studies demonstrating the proline and the glycine content of elastomeric and amyloids proteins, indicate that elastin-like behaviour occurs above a threshold level of combined proline and glycine content; with proline as the primary determinant of elastin-like behaviour^49,50^.

Based upon results obtained by the extensive sequencing of FSP we are presenting here a 3D model for FSP, which was submitted to MD simulations in presence of water, since that the fibres are exposed to air humidity and/or rain under natural conditions. In Guinea *et al*.^51^ studies with *Argiope trifasciata* spider silk, it was demonstrated that high humidity conditions are required for mechanoelastic property response of flagelliform fibre; and that this fibre shares with major ampullate fibre many aspects of their mechanical behaviour.

Modelling of *N. clavipes* FSP was performed for the complete protein, i.e., in the presence of the Non-Repetitive N-terminal Domain (NR-NTD), the three modules of the Central Repetitive Domain (CRD) and the Non-Repetitive C-Terminal Domain (NR-CTD) (Fig. 5). So far only parts of 3D structures of the N- and C-terminal regions of spidroin-1 have been reported^33,39,52,53^. In our own previous studies with *N. clavipes* spider silk, we presented a structural model for spidroin-1 consisting of the N- and C-terminal regions and the repetitive central region composed of a single module of the primary sequence of this protein^15^. The proposed 3D model of FSP indicates the predominance of stretched structures in the solid fibres of the silk protein (like the type I collagen, molecule used as template), presenting a heterogeneous, disordered and randomly oriented structural conformation, even after the simulations by molecular dynamics. In this protein model, the N-terminal region exhibits three complete helices despite the absence of the segment of the sequence at position 110–180 in the N-terminal domain of *N. clavipes* FSP (Fig. S1). The C-terminal domain contains one small helical section, maintained by a disulphide bridge formed by two highly conserved cysteine residues. According to Heim *et al*.^54^ studies with recombinant FSP domains, this disulphide bridge apparently has no influence on secondary or tertiary structure.

MD simulations were performed in a virtual box in presence of water molecules and all model validation parameters revealed that the molecular model was reliable, despite of the predominance of random structures in the solid fibres of the silk. Apparently, the FSP seems to be an intrinsically disordered protein (IDP) or natively unfolded proteins; and this property is a computational challenge for MD simulations, because by definition there are no available 3D structures of the whole molecule that is IDP^55–57^. The structural properties of IDPs are more sensitive to protein-water interactions than those of folded proteins^56^. Protein intrinsic disorder is a state related to protein function^58,59^. Studies have demonstrated that disorder is a metastable state susceptible to the changes in the environment; and that IDPs behaviour is often controlled by PTMs, such as phosphorylation^60^. It is important to mention that our findings pointed a series of PTMs such as phosphorylation and hydroxylation of proline on the FSP; these PTMs could potentially act on mechanoelastic properties and on protein-protein interactions in the web fibres. The data shown in figure S6a indicate that the FSP conformations were stabilized from 6 ns, despite the high abundance of random structures; in the figure S6b,c we can note that FSP exhibited higher molecular radii and higher potential energy in the presence of water. The number of hydrogen bonds in a molecular structure may be considered as an indicator of its stability. FSP contains 600 intramolecular hydrogen bonds in presence of water (Figure S6d), indicating that FSP is structured and stable in presence of water.

The spider silk has a great potential as a biomaterial. To explore this potential, it is needing a better understanding about the structure-function relationship of the proteins present in *N. clavipes* silk fibres; thus, chemical information such as PTMs can provide a basis for the mechanoelastic properties comprehension of silk fibres for biomedical and biotechnology applications. In the present study a large amount of hydroxyproline was observed along the sequence of the FSP, which constitutes the silk of the capture spiral. This silk is known to be highly elastic and the observation of a large amount of hydroxylated proline residues could potentially characterize this elastic property presented by the fibres. Sequence data reported herein may be relevant for the development of novel approaches for the synthetic or recombinant production of novel silk-based spider polymers, forms the basis for understanding previous work and for designing future studies of silks.

## Material and Methods

### Web silk samples

Web silk (radii and spiral) from *N. clavipes* spider was collected at the São Paulo State University at Rio Claro, SP in southeast Brazil. Approximately 25 mg (dry weight) of web silk from 20 orb-web spiders were dissolved in 2 mL of 38 M lithium thiocyanate (LiSCN hydrate) at 25 °C for 2 h under continuous shaking. The web-silk samples were processed as mentionedpreviously by dos Santos-Pinto *et al*.^15^. The protein concentration was estimated by the Bradford assay^61^.

### Scanning electron microscopy

To examine the structural characteristics and the solubilization of the silk fibre surface, a small piece (approximately 5 mm) was placed into the lithium thiocyanate solution. Next, solution aliquots were placed on microscopy stubs and dried on a heating plate at 70 °C for several hours. Thereafter, the stubs were coated with gold in a Cressington sputter coater and then analyzed in a Hitachi, TM-3000 scanning electron microscope at 5–15 kV with a working distance of approximately 8 mm.

### Two-dimensional gel electrophoresis

Silk protein samples (200 μg) were subjected to 2-DE, which was performed without any modification as reported previously by dos Santos-Pinto *et al*.^15^. The gels were stained with *Coomassie Brilliant Blue R-250* (CBB) and thereafter, were scanned for documentation.

### In-gel digestion

The spots of interest were excised from 2-DE gels, de-stained and treated with the following eight proteolytic enzymes: 40 ng/mL of trypsin (Promega, Madison, USA); 50 ng/mL of chymotrypsin (Roche Diagnostics); 40 ng/mL of proteinase 10 (syn.: Thermolysin); 40 ng/µL of Glu-C/V8 protease (Sigma); and 40 ng/µL of subtilisin (Sigma) in different conditions as described previously by dos Santos-Pinto *et al*.^15^ without any modification. Peptide recovering was performed using 0.5% (v/v) formic acid and 0.5% (v/v) formic acid in 30% (v/v) acetonitrile. The extracted peptides were then pooled for nanoLC-ESI-CID/ETD-MS^n^ analysis.

### NanoLC-ESI-CID/ETD-MS^n^

The HPLC used was a Nano-Advance UHPLC system (Bruker, Daltonics, Bremen, Germany) equipped with a PepMap100 C-18 trap column (300 mm × 5 mm) and a PepMap100 C-18 analytical column (75 mm × 150 mm). An Amazon ETD (Bruker Daltonics, Bremen, Germany) equipped with a CaptiveSpray source (Bruker, Daltonics, Bremen, Germany) was used to record the MS^2^ spectra in information-dependent data acquisition (three data-dependent CID MS/MS spectra and three ETD MS/MS spectra) mode over the mass range of m/z 100–3500. At the end, the peak lists from MS/MS data were generated by DataAnalysis 4.1 (Bruker Daltonics), and the spectra were interpreted. The generated spectral data, combining CID MS/MS and ETD MS/MS, were analysed by a MASCOT protein engine search and Modiro^**®**^.

### Database search proteomics

Database searches were performed by using the MASCOT 2.3.02 (Matrix Science, London, UK) against the latest available spider protein sequences deposited in the NCBInr database (http://blast.ncbi.nlm.nih.gov, on July 24, 2017).

Thus, it was selected all 150,235 entries contained in the taxa Araneae for protein identification as reported previously by dos Santos-Pinto *et al*.^15^. Afterwards the identified proteins were subjected to additional filtering by Scaffold 4.3.2 (Proteome Software Inc., Portland, OR) to validate peptide identification. The false discovery rate (FDR) of less than 1%was calculated by requiring significant matches to at least 2 different sequences. Considering the Scaffold Local FDR algorithm, the peptide probability identification was set to a minimum of 99%, while the protein probability identification was set at 95%. PTM searches were performed using Modiro^TM^ - PTM Explorer 1.1 software (Protagen AG, Dortmund, Germany). Using the advanced PTM-explorer search strategies it was possible to perform searches for amino acid substitution and unknown mass shifts. The Modiro software is complementary to the MASCOT software, using already identified sequences and has the advantage that also unknown mass shifts can be handled^62^. A list of 172 common modifications was selected and applied to virtually cleaved and fragmented peptides that were compared with experimentally obtained MS/MS spectra.

### Quantitative proteomics

For the quantification of proteolytic peptides, it was used the spectral counting of the data generated during the mass spectrometry analysis^15,16,63–65^ of the individual digests of *N. clavipes* FSP with trypsin, chymotrypsin, Glu-C/V8 protease, proteinase 10 and subtilisin. The extracted ion chromatograms of each peptide were manually inspected for spectral counting; and the results were normalized dividing the individual counting of each peptide by the lowest counting observed amongst all the peptides.

### Western blotting

In order to check the phosphotyrosine and nitrotyrosine modifications of the FSP, 50 μg of web silk protein extract was loaded onto 1D-SDS-PAGE gels; thereafter, the proteins were transferred onto PVDF membranes (Millipore). The entire procedure was performed as described previously by dos Santos-Pinto *et al*.^15^.

### Secondary structure analysis and molecular modelling

Flagelliform silk protein was subjected to molecular modelling using a restrained-based modelling approach as implemented in the program MODELLER 9v11. Templates for flagelliform silk protein were searched using THREADER v3.5^66^, BLASTp^67^ and FASTA^68^. Template searching by sequential identity using the program BLASTp only found templates that exhibited more than 30% identity to the N-terminal and C-terminal regions. Thus, we decided to search for templates using the THREADER program, which searches according to structural similarity, such that two proteins are considered homologous if they share similar sequences and structures. The output of these tools was formatted and used as input for the MODELLER program, which implements an automated approach for comparative modelling based on the fulfilment of spatial restraints. One thousand models were generated, and the final model was selected based on stereochemical quality and a MODELLER objective function. Images of the three-dimensional structures of the models were generated using PyMOL^34^.

### Molecular dynamics simulations

Molecular dynamics (MD) simulations were performed using the GROMACS 5.1.4 software package^69^, using the force field 43a3^70^ combined with the flexible Simple Point Charge (SPC) water mode^71^. Flagelliform silk protein was subjected to molecular dynamics simulation in a cubic box containing water, and a 1.0-nm minimum distance to the box face was used in all directions. The overall charge on the protein was neutralized, and physiological salt concentrations were simulated using Na^+^ and Cl^-^ counter-ions. During the simulations, the lengths of the bonds within the protein were constrained under the conditions set by LINCS^72^ and SETTLE^73^ for water geometry. In the initial MD simulations, all hydrogen atoms, ions, and water molecules were subjected to 500 steps of energy minimization to remove close van der Waals contacts. The water system was then subjected to a short MD simulation with position restraints for 1,000 pico-seconds (ps). The final MD simulations were performed under the same conditions, except that the position restraints were removed for 10,000 ps. Energy minimization and MD were performed under periodic boundary conditions. Simulation was accomplished in the isothermal-isobaric ensemble at 300 K using temperature coupling^74^ and a constant pressure of 1 atm with isotropic molecule-based scaling^75^. Temperature and pressure were modulated using coupling techniques^74^ with coupling and isothermal compressibility constants of 0.01 ps (solvent and protein) and 6.5 × 10−5 bar^−1^, respectively. Electrostatic interactions among non-ligand atoms were evaluated using the particle-mesh Ewald method^76^. Cut-off distances for the calculation of the Coulomb and van der Waals interactions were 1.0 and 1.4 nm, respectively. Molecular visualization was performed in the graphical environments VMD - Visual Molecular Dynamics^77^ and PyMOL^34^.

## Electronic supplementary material

Supplementary information
