# Supplementary material for: Spider silk proteome provides insight into the structural characterization of Nephila clavipes flagelliform spidroin
Source: Sci Rep. 2018 Oct 2;8:14674. doi: 10.1038/s41598-018-33068-9 (PMC6168590; doi:10.1038/s41598-018-33068-9)
Supplement: Supplementary file 1 — Supplementary information [file 41598_2018_33068_MOESM1_ESM.pdf]

Supplementary information

FIGURES and TABLES

Spider silk proteome provides insight into the structural characterization of *Nephila clavipes* flagelliform spidroin

José Roberto Aparecido dos Santos-Pinto[1], Helen Andrade Arcuri[1], Franciele Grego Esteves[1], Mario Sergio Palma*[,1] and Gert Lubec*[,2]

[1]Center of the Study of Social Insects, Department of Biology, Institute of Biosciences of Rio Claro, São Paulo State University, Rio Claro, SP, Brazil 13500; [2] Paracelsus Medical University, A 5020 Salzburg, Austria

*Corresponding authors. Prof. Dr. Gert Lubec, Paracelsus Medical University, A 5020 Salzburg, Austria, E-mail: gert.lubec@lubeclab.com, and Prof. Dr. Mario Sergio Palma, CEIS-IBRC-UNESP, Av. 24 A, nº 1515, Bela Vista - Rio Claro, SP, Brazil, CEP 13506-900, E-mail: mspalma@rc.unesp.br

Supplementary Figure S1.

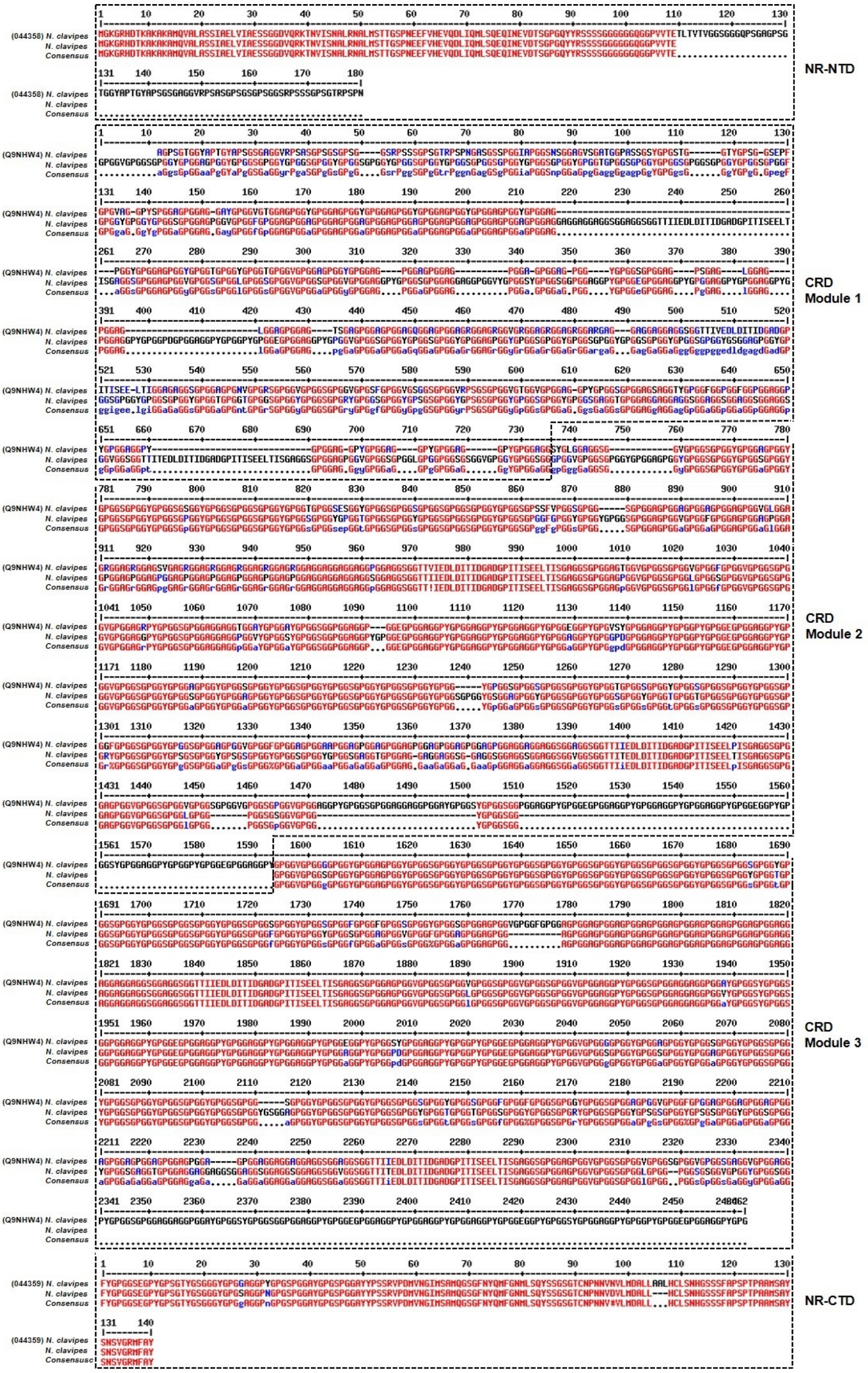

Figure S1. Multiple alignment of sequence of all the proteolytic fragments obtained for *N. clavipes* flagelliform silk protein with the sequences of the protein fragment from *N. clavipes* (accession number O44358 / NR-NTD - non-repetitive N-terminal domain), *N. clavipes* (accession number Q9NHW4 / CRD - central repetitive domain) and *N. clavipes* (accession number O44359 / NR-CTD - non-repetitive C-terminal domain). Red - high sequence conservation; Blue - low sequence conservation. It was performed by Multialign Server (http://multalin.toulouse.inra.fr/multalin/).

Supplementary Figures S2-S5. Representative mass spectra of the identified post-translational modifications of flagelliform silk protein from the flagelliform silk produced by *N. clavipes.*

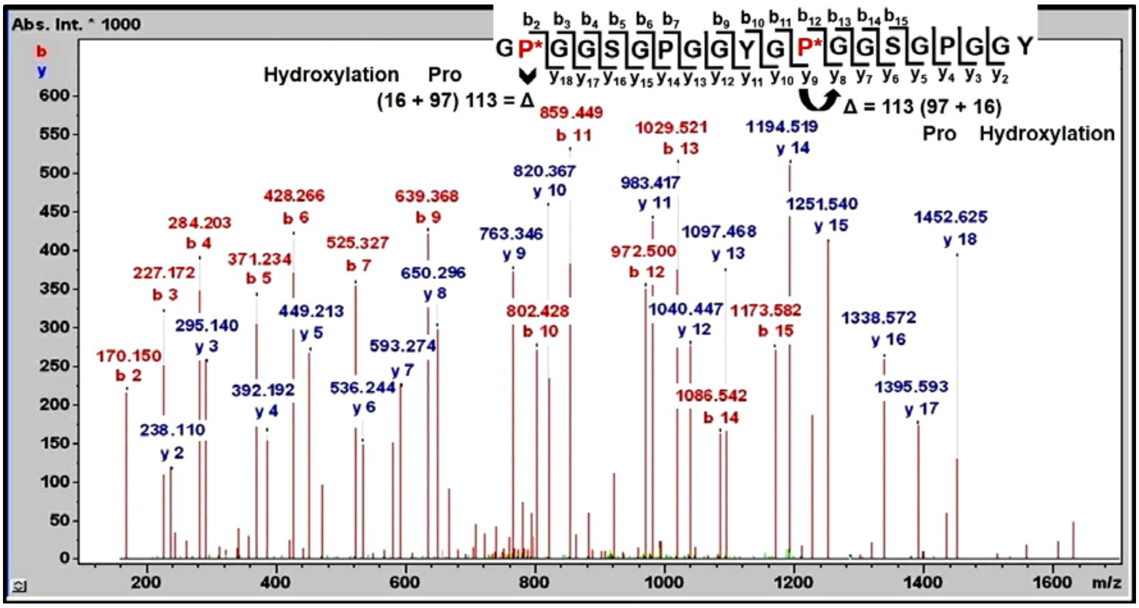

Figure S2. Representative CID spectrum of chymotryptic peptide GP*GGSGPGGYGP*GGSGPGGY (135-154, 155-174, 578-597, 598-617, 628-647, 870-889, 890-909, 1313-1332, 1333-1352, 1363-1382, 1605-1624, 1625-1644, 2048-2067, 2068-2087, 2098-2117), selecting the m/z 812.350 $[M + 2H]^{2+}$, as precursor ion; and showing the P136, P146, P156, P166, P579, P589, P599, P609, P629, P639, P871, P881, P891, P901, P1314, P1324, P1334, P1344, P1364, P1374, P1606, P1616, P1626, P1636, P2049, P2059, P2069, P2079, P2099 and P2109 hydroxyproline sites observed on the flagelliform silk protein.

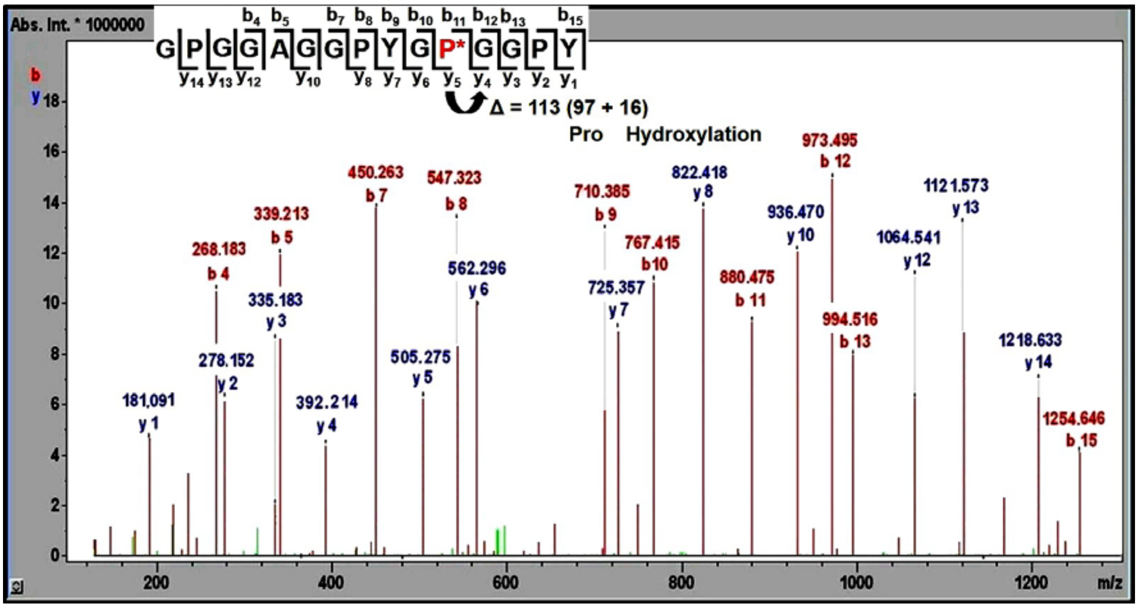

Figure S3. Representative CID spectrum of chymotryptic peptide GPGGAGGPYGP*GGPY (514-528, 1249-1263, 1984-1998), selecting the m/z 638.800 $[M + 2H]^{2+}$, as precursor ion; and showing the P524, P1259 and P1994 hydroxyproline sites observed on the flagelliform silk protein.

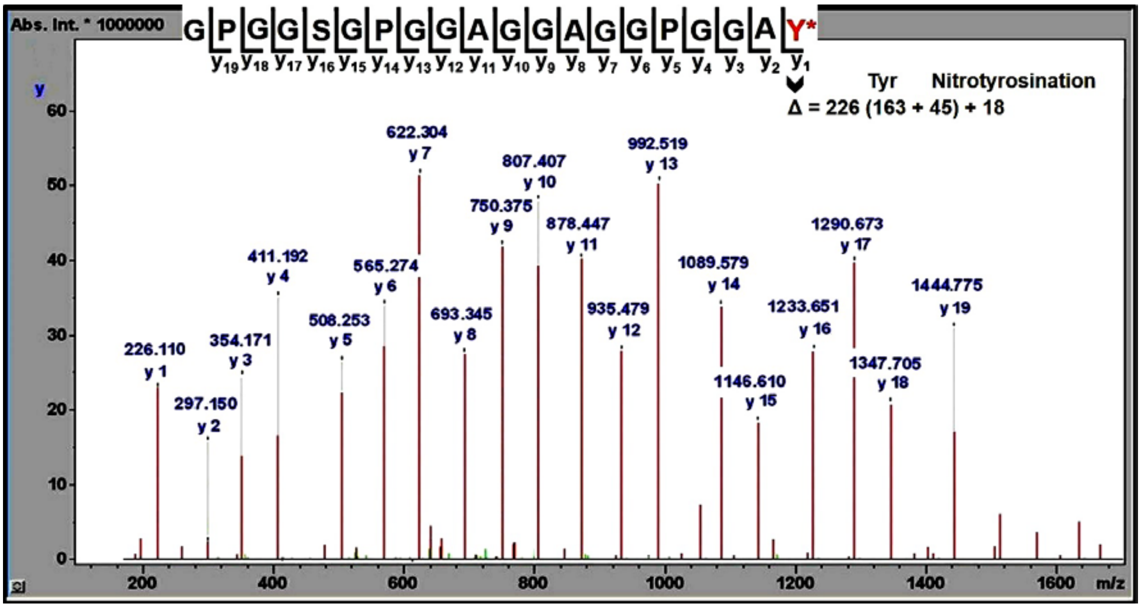

Figure S4. Representative CID spectrum of chymotryptic peptide GPGGSGPGGAGGAGGPGGAY* (426-445, 1161-1180, 1896-1915), selecting the m/z 751.880 $[M + 2H]^{2+}$, as precursor ion; and showing the Y445, Y1180 and Y1915 nitrotyrosine sites observed on the flagelliform silk protein.

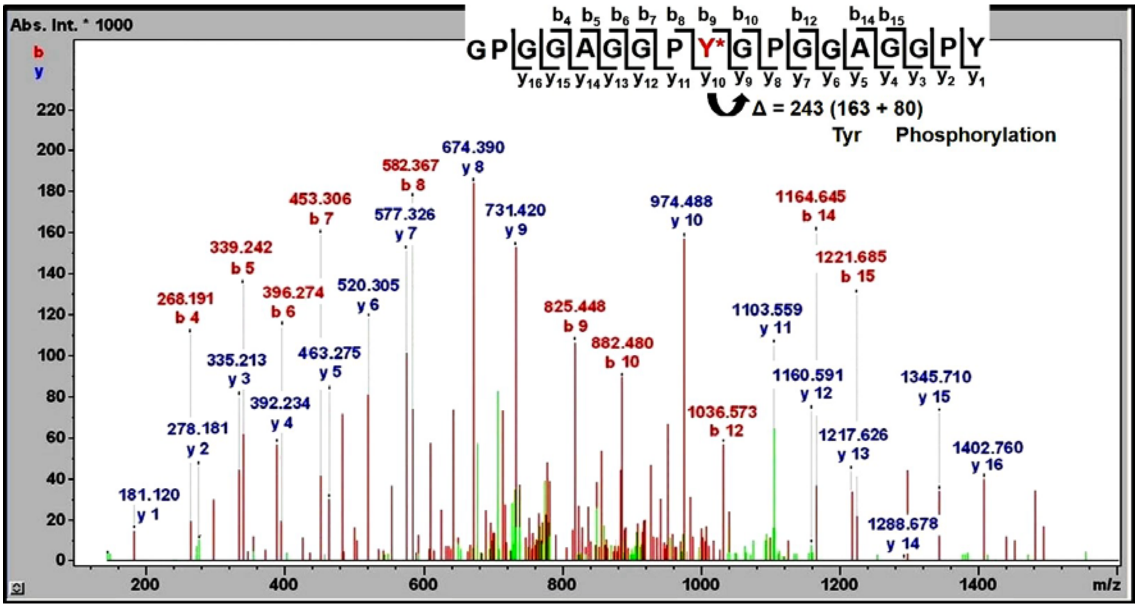

Figure S5. Representative CID spectrum of chymotryptic peptide GPGGAGGP*Y*GPGGAGGPY (481-498, 1216-1233, 1951-1968), selecting the m/z 779.390 $[M + 2H]^{2+}$, as precursor ion; and showing the P488, P1223 and P1958 dihydroxyproline sites and Y489, Y1224 and Y1959 phosphotyrosine sites observed on the flagelliform silk protein.

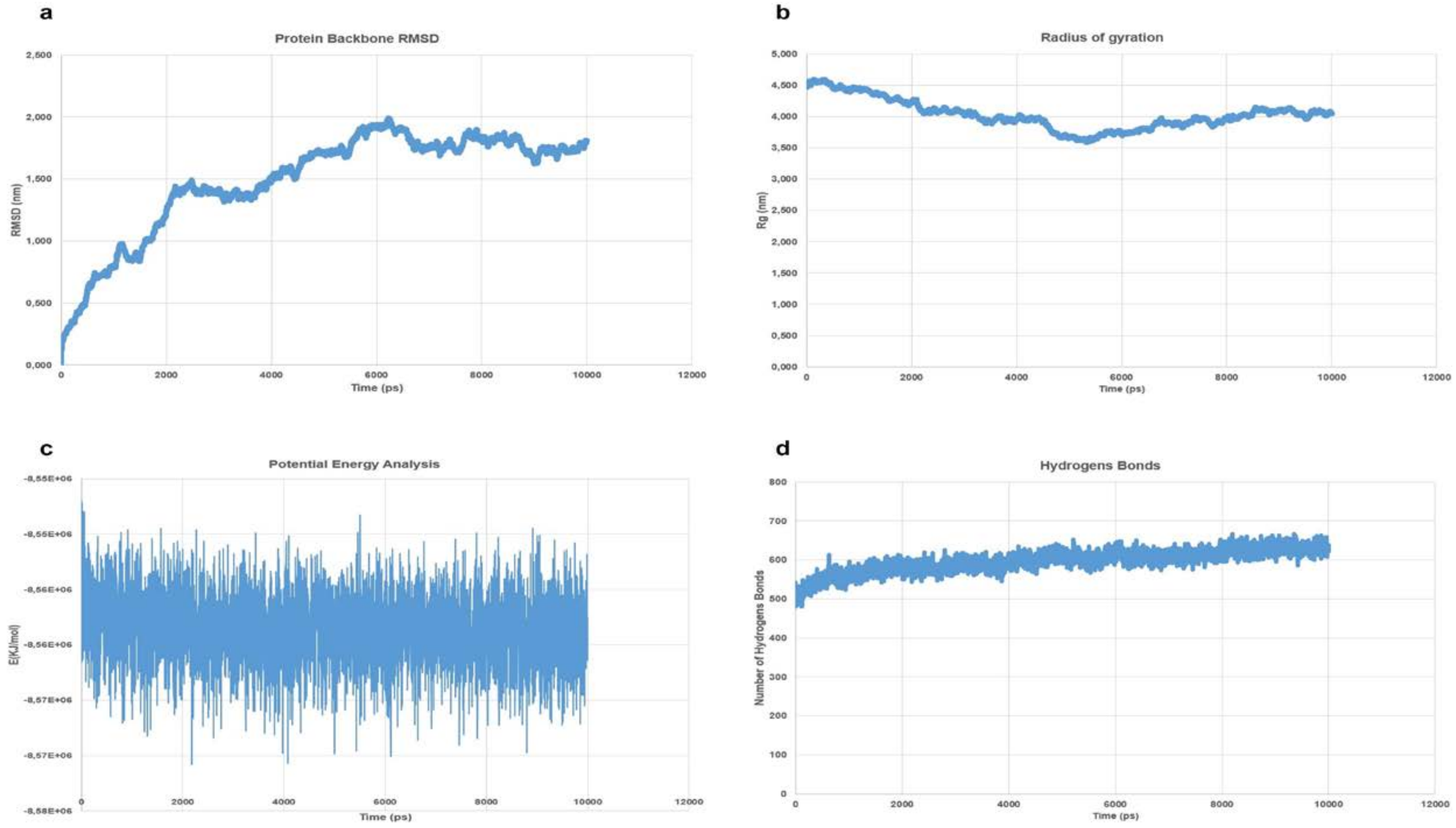

Figure S6. Structural analysis of three-dimensional molecular models of flagelliform silk protein after 10 ns of molecular dynamics simulation in water. **a -** Protein backbone RMSD. **b -** Radius of gyration. **c -** Potential energy analysis. **d -** Hydrogen bonds.

Supplementary Tables S1-S3.

Table S1. Amino acid sequence of flagelliform silk protein from the web silk produced by *N. clavipes* after in-gel digestion using various proteolytic enzymes. Spot number, accession number, protein name, enzyme used, amino acid position, observed m/z, experimental mass, theoretical mass, difference between experimental mass and theoretical mass, number of missed cleavage sites, peptide sequences, PTMs, fragmentation method and MASCOT íon scores are listed for all identified peptides.

Spot	Accession number	Protein	Enzyme	Amino acid position	Observed m/z (charge)	Mr expect	Mr cal	Delta	Missed cleavage	Peptide Sequence	PTMs / Fragmentation method	MASCOT íons score
1	O44358	Flageliform silk protein	Trypsin	14-38	834.7257 (+3)	2501.1553	2501.2795	-0.1243	0	K.AMQVALASSIAELVIAESSGGDVQR.K	CID + ETD	97
1	O44358	Flageliform silk protein	Trypsin	39-48	558.3191 (+2)	1114.6236	1114.6458	-0.0222	1	R.KTNVISNALR.N	CID + ETD	24
1	O44358	Flageliform silk protein	Trypsin	40-48	494.2738 (+2)	986.5330	986.5509	-0.0178	0	K.TNVISNALR.N	CID	43
1	O44358	Flageliform silk protein	Glu-C	26-30	544.3100 (+1)	543.3027	543.3268	-0.0241	0	E.LVIAE.S	CID	29
1	O44358	Flageliform silk protein	Glu-C	31-61	1078.7969 (+3)	3233.3689	3233.5582	-0.1893	1	E.SSGGDVQRKTNVISNALRNALMSTTGSPNEE.F	CID + ETD	39
1	O44358	Flageliform silk protein	Glu-C	66-80	894.5055 (+2)	1786.9964	1786.8771	0.1194	1	E.VQDLIQMLSQEQINE.V	CID	87
1	O44358	Flageliform silk protein	Glu-C	81-109	900.7024 (+3)	2699.0854	2699.2060	-0.1206	0	E.VDTSGPGQYYRSSSSGGGGGGGGGPVVTE.T	CID + ETD	40
1	O44359	Flageliform silk protein	Chymotrypsin	1-25	971.9553 (+2)	1941.8960	1941.8606	0.0354	1	Y.GPGGVGPGGSGPGGYGPGGAGPGGY.G	CID	97
1	O44359	Flageliform silk protein	Chymotrypsin	26-45; 36-55; 46-65; 56-75; 439-458; 469-488; 479-498; 489-508; 519-538	804.9257 (+2)	1607.8368	1607.7044	0.1324	1	Y.GPGGSGPGGYGPGGSGPGGY.G	CID	94
1	O44359	Flagelliform silk protein	Chymotrypsin	26 - 35; 36 - 45; 46 - 55; 56 - 65; 469 - 478; 479 -	411.2100 (+2)	820.4200	820.422	0.002	2	Y.GP*GGSGPGGY.G	Hydroxylation (P27, P37, P47, P57, P470, P480,	100

				488; 489 - 498; 519 – 528							P490, P520) / CID	
1	O44359	Flageliform silk protein	Chymotrypsin	121-140	796.3983 (+2)	1590.7820	1590.6700	0.1121	1	Y.GPGGSGPGGYGPGGSGPGGY.G	CID	108
1	O44359	Flageliform silk protein	Chymotrypsin	317-336	743.3921 (+2)	1484.7696	1484.6645	0.1052	0	Y.GPGGSGPGGAGGAGGPGGA*Y.G	Ala->Val (A335) / CID	106
1	O44359	Flageliform silk protein	Chymotrypsin	317-336	751.8770 (+2)	1501.7394	1501.6182	0.1212	0	Y.GPGGSGPGGAGGAGGPGGAY.G	CID	51
1	O44359	Flageliform silk protein	Chymotrypsin	337-357	831.9373 (+2)	1661.8600	1661.7071	0.1530	1	Y.GPGGSYGPGGSGGPGGAGGPY.G	CID	63
1	O44359	Flageliform silk protein	Chymotrypsin	343-371	770.3529 (+3)	2308.0369	2307.8869	0.1500	1	Y.GPGGSGGPGGAGGPYGPGGEGPGGAGGPY.G	CID + ETD	53
1	O44359	Flageliform silk protein	Chymotrypsin	358 - 371; 420 - 433	565.3246 (+2)	1128.6346	1128.4836	0.1510	0	Y.GPGGEGPGGAGGPY.G	CID + ETD	63
1	O44359	Flageliform silk protein	Chymotrypsin	358-380	930.4636 (+2)	1858.9126	1858.8314	0.0812	1	Y.GPGGEGPGGAGGPYGPGGAGGPY.G	CID	81
1	O44359	Flageliform silk protein	Chymotrypsin	372-389	779.3906 (+2)	1556.7666	1556.5933	0.1733	1	Y.GPGGAGGP*Y*GPGGAGGPY.G	Dihydroxylation (P379); Phospho (Y380) / CID	30
1	O44359	Flageliform silk protein	Chymotrypsin	381-398	752.4004 (+2)	1502.7862	1502.6427	0.1436	1	Y.GPGGAGGPYGPGGEGGPY.G	CID	82
1	O44359	Flageliform silk protein	Chymotrypsin	390-398	732.3884 (+1)	731.3811	731.3239	0.0572	0	Y.GPGGE*GGPY.G	Glu->Ala (E394) / CID	46
1	O44359	Flageliform silk protein	Chymotrypsin	399-419	886.9786 (+2)	1771.9426	1771.7802	0.1624	2	Y.GPGGPY*GPGGAGGPYGPGGPY.G	Tyr->Asp (Y404) / CID	69
1	O44359	Flageliform silk protein	Chymotrypsin	414-433	829.4463 (+2)	1656.8780	1656.7169	0.1612	1	Y.GPGGPYGPGGEGPGGAGGPY.G	CID + ETD	119
1	O44359	Flageliform silk protein	Chymotrypsin	509-528	804.9257 (+2)	1607.8368	1607.6601	0.1767	1	Y.GSGGAGPGGYGPGGSGPGGY.G	CID	43
1	O44359	Flageliform silk protein	Proteinase 10	25-44; 478-497; 518-537;	796.300 (+2)	1590.585	1590.670	-0.0845	0	G.YGPGGSGPGGYGPGGSGPGG.Y	CID + ETD	48

1	O44359	Flageliform silk protein	Proteinase 10	109-129	786.810 (+2)	1571.605	1571.660	-0.0547	0	G.GSGPGGSGPGGYGPGGSGPGG.F	CID + ETD	70
1	O44359	Flageliform silk protein	Proteinase 10	352-374	921.920 (+2)	1841.825	1841.796	0.0285	0	G.GAGGPYGPGGEGPGGAGGPYGPG.G	CID	62
1	O44359	Flageliform silk protein	Proteinase 10	408-427	829.370 (+2)	1656.725	1656.716	0.0086	0	G.GAGGPYGPGGPYGPGGEGPG.G	CID + ETD	64
1	O44359	Flageliform silk protein	Proteinase 10	410-427	765.320 (+2)	1528.625	1528.658	-0.0329	0	A.GGPYGPGGPYGPGGEGPG.G	CID + ETD	68
1	O44359	Flageliform silk protein	Subtilisin	6-15	805.3023 (+1)	804.2950	804.3403	-0.0452	0	V.GPGGSGPGGY.G	CID	39
1	O44359	Flageliform silk protein	Subtilisin	234-242	918.3370 (+1)	917.3297	917.4706	-0.1409	0	S.GGTTIIEDL.D	CID	35
1	O44359	Flageliform silk protein	Subtilisin	281-290; 291-300; 301-310; 704-713	741.3011 (+1)	740.2938	740.3454	-0.0515	0	G.GSGPGGVGPG.G	CID	51
2	O44358	Flageliform silk protein	Glu-C	26-30	544.3056 (+1)	543.2983	543.3268	-0.0285	0	E.LVIAE.S	CID	19
2	O44358	Flageliform silk protein	Glu-C	31-60	1078.4963 (+3)	3232.4671	3232.5742	-0.1071	0	E.SSGGDVQRKTNVISNALRNALMSTTGSPNE.E	CID + ETD	39
2	O44358	Flageliform silk protein	Glu-C	31-65	941.4182 (+4)	3761.6437	3761.8045	-0.1608	2	E.SSGGDVQRKTNVISNALRNALM*STTGSPNEEFVHE.V	Oxidation (M52) / ETD	31
2	O44358	Flageliform silk protein	Glu-C	66-80	894.4152 (+2)	1786.8158	1786.8771	-0.0612	1	E.VQDLIQMLSQEQINE.V	CID	87
2	O44358	Flageliform silk protein	Glu-C	81-109	900.6979 (+3)	2699.0719	2699.2060	-0.1341	0	E.VDTSGPGQYYRSSSSGGGGGGQGGPVVTE.T	CID + ETD	87
2	O44358	Flageliform silk protein	Trypsin	39-48	372.5829 (+3)	1114.7269	1114.6458	0.0810	1	R.KTNVISNALR.N	CID + ETD	32
2	O44358	Flageliform silk protein	Trypsin	40-48	494.2347 (+2)	986.4548	986.5509	-0.0960	0	K.TNVISNALR.N	CID + ETD	35

2	O44359	Flageliform silk protein	Chymotrypsin	1 - 15; 434 - 448	586.7690 (+2)	1171.5222	1171.5211	-0.0011	0	GPGGVGPGGSGPGGY.G	CID	58
2	O44359	Flageliform silk protein	Chymotrypsin	1-25	648.2941 (+3)	1941.8646	1941.8623	0.0023	1	GPGGVGPGGSGPGGYGPGGAGPGGY.G	CID + ETD	36
2	O44359	Flageliform silk protein	Chymotrypsin	1-45	1172.5101 (+3)	3514.5294	3514.5247	0.0047	3	GPGGVGPGGSGPGGYGPGGAGPGGYGPGGSGP GGYGPGGSGPGGY.G	CID	33
2	O44359	Flageliform silk protein	Chymotrypsin	16-35; 459-478	788.3410 (+2)	1574.6808	1574.6754	0.0054	1	Y.GPGGAGPGGYGPGGSGPGGY.G	CID	36
2	O44359	Flageliform silk protein	Chymotrypsin	26 - 45; 36 - 55; 46 - 65; 56 - 75; 469 - 488; 479 - 498; 489 - 508; 519 - 538	796.3401 (+2)	1590.6707	1590.6706	0.0001	1	Y.GPGGSGPGGYGPGGSGPGGY.G	CID	53
2	O44359	Flageliform silk protein	Chymotrypsin	76-90	618.7610 (+2)	1235.5162	1235.5229	-0.0067	1	Y.GPGGYGPGGSGPGGY.G	CID	31
2	O44359	Flageliform silk protein	Chymotrypsin	106-120	580.7501 (+2)	1159.4890	1159.4880	-0.0010	0	Y.GPGGSGPGGSGPGGY.G	CID	34
2	O44359	Flageliform silk protein	Chymotrypsin	106-130	644.2820 (+3)	1929.8153	1929.8207	-0.0054	1	Y.GPGGSGPGGSGPGGYGPGGSGPGGF.G	CID	38
2	O44359	Flageliform silk protein	Chymotrypsin	260-316	1059.7501 (+4)	4234.9727	4234.9614	0.0113	0	L.TISGAGGSGPGGAGPGGVGPGGSGPGGVGPGG SGPGGVGPGGSGPGGVGPGGAGGPY.G	ETD	40
2	O44359	Flageliform silk protein	Chymotrypsin	317-336	729.3212 (+2)	1456.6380	1456.6331	0.0049	0	Y.GPGGSGPGGAGGAGGPGGAY.G	CID	32
2	O44359	Flageliform silk protein	Chymotrypsin	337-357	831.8520 (+2)	1661.7033	1661.7059	-0.0026	1	Y.GPGGSYGPGGSGGPGGAGGPY.G	CID + ETD	64
2	O44359	Flageliform silk protein	Chymotrypsin	343-357	572.7510 (+2)	1143.4902	1143.4906	-0.0004	0	Y.GPGGSGGPGGAGGPY.G	CID	36
2	O44359	Flageliform silk protein	Chymotrypsin	343-371	752.3200 (+3)	2253.9517	2253.9644	-0.0127	1	Y.GPGGSGGPGGAGGPYGPGGEGPGGAGGPY.G	CID + ETD	30
2	O44359	Flageliform silk protein	Chymotrypsin	343-380	990.0901 (+3)	2967.2791	2967.2830	-0.0039	2	Y.GPGGSGGPGGAGGPYGPGGEGPGGAGGPYGP GGAGGPY.G	CID + ETD	42
2	O44359	Flageliform silk protein	Chymotrypsin	358 - 371; 420 - 433	565.2411 (+2)	1128.4804	1128.4808	-0.0004	0	Y.GPGGEGPGGAGGPY.G	CID	68

2	O44359	Flageliform silk protein	Chymotrypsin	358-380	921.9002 (+2)	1841.8024	1841.7982	0.0042	1	Y.GPGGEGPGGAGGPYGPGGAGGPY.G	CID	48
2	O44359	Flageliform silk protein	Chymotrypsin	372 - 380; 381 - 389; 405 - 413; 791 - 799	366.6620 (+2)	731.3209	731.3201	0.0008	0	Y.GPGGAGGPY.G	CID	44
2	O44359	Flageliform silk protein	Chymotrypsin	372-389	763.4110 (+2)	1524.8230	1524.8320	-0.0009	1	Y.GPGGAGGPYGPGGAGGPY*.G	Phospho (Y389) / CID	66
2	O44359	Flageliform silk protein	Chymotrypsin	372-398	1108.9822 (+2)	2215.9609	2215.9541	0.0068	2	Y.GPGGAGGPYGPGGAGGPYGPGGEGGPY.G	CID	36
2	O44359	Flageliform silk protein	Chymotrypsin	381-398	752.3210 (+2)	1502.6351	1502.6408	-0.0057	1	Y.GPGGAGGPYGPGGEGGPY.G	CID + ETD	50
2	O44359	Flageliform silk protein	Chymotrypsin	405-419	630.7800 (+2)	1259.5503	1259.5509	-0.0006	1	Y.GPGGAGGPYGPGGPY.G	CID	50
2	O44359	Flageliform silk protein	Chymotrypsin	405-433	1186.0210 (+2)	2370.0330	2370.0301	0.0029	2	Y.GPGGAGGPYGPGGPYGPGGEGPGGAGGPY.G	CID	31
2	O44359	Flageliform silk protein	Chymotrypsin	414-433	829.3613 (+2)	1656.7101	1656.7107	-0.0006	1	Y.GPGGPYGPGGEGPGGAGGPY.G	CID + ETD	52
2	O44359	Flageliform silk protein	Chymotrypsin	420-448	1142.0112 (+2)	2282.0198	2281.9923	0.0175	1	Y.GPGGEGPGGAGGPYGPGGVGPGGSGPGGY.G	CID	42
2	O44359	Flageliform silk protein	Chymotrypsin	434-458	979.9421 (+2)	1957.8707	1957.8552	0.0155	1	Y.GPGGVGPGGSGPGGYGPGGSGPGGY.G	CID + ETD	40
2	O44359	Flageliform silk protein	Chymotrypsin	609-665	1042.7031 (+4)	4166.8009	4166.7361	0.0648	0	Y.GPGGSGAGGTGPGGAGGAGGAGGSGGAGGSG GAGGSGGAGGSGGVGGSGGTTITEDL.D	ETD	38
2	O44359	Flageliform silk protein	Chymotrypsin	746-760	621.7623 (+2)	1241.5111	1241.4700	0.0411	0	Y.GPGGSGSGGVGPGGY.G	CID	33
2	O44359	Flageliform silk protein	Chymotrypsin	746-768	889.8900 (+2)	1777.7646	1777.7624	0.0022	1	Y.GPGGSGSGGVGPGGYGPGGSGGF.Y	CID + ETD	40
2	O44359	Flageliform silk protein	Chymotrypsin	746-769	971.4201 (+2)	1940.8260	1940.8293	-0.0033	2	Y.GPGGSGSGGVGPGGYGPGGSGGFY.G	CID	35
2	O44359	Flageliform silk protein	Chymotrypsin	770-784	691.7921 (+2)	1381.5714	1381.5756	-0.0012	1	Y.GPGGSEGPYGPSGTY.G	CID + ETD	32
2	O44359	Flageliform silk protein	Chymotrypsin	809-844	1024.1731 (+4)	4092.6605	4092.5010	0.1595	3	Y.GPGSPGGAYYPSSRVPDMVNGIMSAMQGSGFNY QM*F.G	Oxidation (M843) / ETD	38

2	O44359	Flageliform silk protein	Proteinase 10	4-14	798.340 (+1)	797.332	797.366	-0.0341	0	G.GVGPGGSGPGG.Y	CID	31
2	O44359	Flageliform silk protein	Proteinase 10	5-14	741.340 (+1)	740.332	740.345	-0.0126	0	G.VGPGGSGPGG.Y	CID	36
2	O44359	Flageliform silk protein	Proteinase 10	5-18	1115.410 (+1)	1114.402	1114.504	-0.1017	0	G.VGPGGSGPGGYGPG.G	CID	39
2	O44359	Flageliform silk protein	Proteinase 10	5-19	586.760 (+2)	1171.505	1171.525	-0.0204	0	G.VGPGGSGPGGYGPGG.A	CID + ETD	35
2	O44359	Flageliform silk protein	Proteinase 10	5-24	756.330 (+2)	1510.645	1510.680	-0.0347	0	G.VGPGGSGPGGYGPGGAGPGG.Y	CID	70
2	O44359	Flageliform silk protein	Proteinase 10	5-28	943.410 (+2)	1884.805	1884.839	-0.0337	0	G.VGPGGSGPGGYGPGGAGPGGYGPG.G	CID + ETD	56
2	O44359	Flageliform silk protein	Proteinase 10	15-34; 458-477	788.320 (+2)	1574.625	1574.675	-0.0496	0	G.YGPGGAGPGGYGPGGSGPGG.Y	CID + ETD	34
2	O44359	Flageliform silk protein	Proteinase 10	20-38	759.790 (+2)	1517.565	1517.653	-0.0881	0	G.AGPGGYGPGGSGPGGYGPG.G	CID	45
2	O44359	Flageliform silk protein	Proteinase 10	20-54	906.360 (+3)	2716.058	2716.153	-0.0958	0	G.AGPGGYGPGGSGPGGYGPGGSGPGGYGPGGS GPGG.Y	CID + ETD	31
2	O44359	Flageliform silk protein	Proteinase 10	24-44; 477-497; 517-537	824.820 (+2)	1647.625	1647.691	-0.0660	0	G.GYGPGGSGPGGYGPGGSGPGG.Y	CID	71
2	O44359	Flageliform silk protein	Proteinase 10	24-48	1011.880 (+2)	2021.745	2021.850	-0.1050	0	G.GYGPGGSGPGGYGPGGSGPGGYGPG.G	CID	97
2	O44359	Flageliform silk protein	Proteinase 10	24-54	812.320 (+3)	2433.938	2434.021	-0.0829	0	G.GYGPGGSGPGGYGPGGSGPGGYGPGGSGPGG. Y	ETD	29
2	O44359	Flageliform silk protein	Proteinase 10	24-58	937.080 (+3)	2808.218	2808.180	0.0380	0	G.GYGPGGSGPGGYGPGGSGPGGYGPGGSGPGG YGPG.G	CID + ETD	47
2	O44359	Flageliform silk protein	Proteinase 10	25-38	590.220 (+2)	1178.425	1178.499	-0.0738	0	G.YGPGGSGPGGYGPG.G	CID	39
2	O44359	Flageliform silk protein	Proteinase 10	25-43	767.810 (+2)	1533.605	1533.648	-0.0430	0	G.YGPGGSGPGGYGPGGSGPG.G	CID + ETD	84
2	O44359	Flageliform silk protein	Proteinase 10	25-48	983.380 (+2)	1964.745	1964.829	-0.0835	0	G.YGPGGSGPGGYGPGGSGPGGYGPG.G	CID + ETD	106

2	O44359	Flageliform silk protein	Proteinase 10	25-49	1011.890 (+2)	2021.765	2021.850	-0.0850	0	G.YGPGGSGPGGYGPGGSGPGGYGPGG.S	CID + ETD	46
2	O44359	Flageliform silk protein	Proteinase 10	25-53	774.280 (+3)	2319.818	2319.978	-0.1600	0	G.YGPGGSGPGGYGPGGSGPGGYGPGGSGPG.G	ETD	66
2	O44359	Flageliform silk protein	Proteinase 10	25-54	793.310 (+3)	2376.908	2376.999	-0.0915	0	G.YGPGGSGPGGYGPGGSGPGGYGPGGSGPGG.Y	CID + ETD	55
2	O44359	Flageliform silk protein	Proteinase 10	25-63	1036.380 (+3)	3106.118	3106.307	-0.1897	0	G.YGPGGSGPGGYGPGGSGPGGYGPGGSGPGGY GPGGSGPG.G	CID	74
2	O44359	Flageliform silk protein	Proteinase 10	29-44	609.230 (+2)	1216.445	1216.510	-0.0655	0	G.GSGPGGYGPGGSGPGG.Y	CID	35
2	O44359	Flageliform silk protein	Proteinase 10	29-53	973.870 (+2)	1945.725	1945.819	-0.0937	0	G.GSGPGGYGPGGSGPGGYGPGGSGPG.G	CID	109
2	O44359	Flageliform silk protein	Proteinase 10	29-58	793.340 (+3)	2376.998	2376.999	-0.0015	0	G.GSGPGGYGPGGSGPGGYGPGGSGPGGYGPG.G	CID + ETD	37
2	O44359	Flageliform silk protein	Proteinase 10	29-63	911.690 (+3)	2732.048	2732.148	-0.1007	0	G.GSGPGGYGPGGSGPGGYGPGGSGPGGYGPGG SGPG.G	CID + ETD	71
2	O44359	Flageliform silk protein	Proteinase 10	29-64	930.690 (+3)	2789.048	2789.170	-0.1221	0	G.GSGPGGYGPGGSGPGGYGPGGSGPGGYGPGG SGPGG.Y	ETD	52
2	O44359	Flageliform silk protein	Proteinase 10	30-64	911.690 (+3)	2732.048	2732.148	-0.1007	0	G.SGPGGYGPGGSGPGGYGPGGSGPGGYGPGGS GPGG.Y	CID + ETD	48
2	O44359	Flageliform silk protein	Proteinase 10	55-88	944.700 (+3)	2831.078	2831.125	-0.0468	0	G.YGPGGSGPGGYGPGGSGPGGYGPGGYGPGGS GPG.G	CID + ETD	50
2	O44359	Flageliform silk protein	Proteinase 10	92-114	910.890 (+2)	1819.765	1819.716	0.0491	0	G.PGGTGPGGSGPGGYGPGGSGPGG.S	CID	26
2	O44359	Flageliform silk protein	Proteinase 10	99-123	973.880 (+2)	1945.745	1945.819	-0.0737	0	G.GSGPGGYGPGGSGPGGSGPGGYGPG.G	CID	45
2	O44359	Flageliform silk protein	Proteinase 10	99-124	1002.370 (+2)	2002.725	2002.840	-0.1152	0	G.GSGPGGYGPGGSGPGGSGPGGYGPGG.S	CID	65
2	O44359	Flageliform silk protein	Proteinase 10	99-129	786.980 (+3)	2357.918	2357.989	-0.0717	0	G.GSGPGGYGPGGSGPGGSGPGGYGPGGSGPGG. F	CID + ETD	39
2	O44359	Flageliform silk protein	Proteinase 10	100-119	758.290 (+2)	1514.565	1514.638	-0.0732	0	G.SGPGGYGPGGSGPGGSGPGG.Y	CID	55

2	O44359	Flageliform silk protein	Proteinase 10	104-123	796.300 (+2)	1590.585	1590.670	-0.0845	0	G.GYGPGGSGPGGSGPGGYGPG.G	CID	74
2	O44359	Flageliform silk protein	Proteinase 10	104-129	1002.360 (+2)	2002.705	2002.840	-0.1352	0	G.GYGPGGSGPGGSGPGGYGPGGSGPGG.F	CID	102
2	O44359	Flageliform silk protein	Proteinase 10	105-129	973.870 (+2)	1945.725	1945.819	-0.0937	0	G.YGPGGSGPGGSGPGGYGPGGSGPGG.F	CID + ETD	82
2	O44359	Flageliform silk protein	Proteinase 10	109-129	786.840 (+2)	1571.665	1571.660	0.0053	0	G.GSGPGGSGPGGYGPGGSGPGG.F	CID	54
2	O44359	Flageliform silk protein	Proteinase 10	116-149	930.700 (+3)	2789.078	2789.054	0.0236	0	S.GPGGYGPGGSGPGGFGPGGSGPGGYGPGGSGPGG.A	CID + ETD	29
2	O44359	Flageliform silk protein	Proteinase 10	125-149	965.870 (+2)	1929.725	1929.824	-0.0988	0	G.SGPGGFGPGGSGPGGYGPGGSGPGG.A	CID	100
2	O44359	Flageliform silk protein	Proteinase 10	140-154	1144.500 (+1)	1143.492	1143.494	-0.0018	0	G.YGPGGSGPGGAGPGG.V	CID	40
2	O44359	Flageliform silk protein	Proteinase 10	150-159	725.320 (+1)	724.312	724.350	-0.0377	0	G.AGPGGVGPGG.F	CID	27
2	O44359	Flageliform silk protein	Proteinase 10	160-187	1008.410 (+2)	2014.805	2014.924	-0.1192	0	G.FGPGGAGPGGAGPGGAGPGGAGPGGAGP.G	CID + ETD	25
2	O44359	Flageliform silk protein	Proteinase 10	179-238	1023.950 (+4)	4091.770	4091.841	-0.0707	0	G.GAGPGGAGPGGAGPGGAGPGGAGPGGAGPGGAGPGGAGGAGGAGGAGGSGGAGGSGGTTI.I	ETD	35
2	O44359	Flageliform silk protein	Proteinase 10	201-230	686.610 (+3)	2056.808	2056.834	-0.0267	0	A.GPGGAGPGGAGPGGAGGAGGAGGAGGSGGA.G	CID + ETD	27
2	O44359	Flageliform silk protein	Proteinase 10	228-255	916.050 (+3)	2745.128	2745.203	-0.0753	0	S.GGAGGSGGTTIIEDLDITIDGADGPITI.S	CID + ETD	38
2	O44359	Flageliform silk protein	Proteinase 10	246-254	858.430 (+1)	857.422	857.413	0.0097	0	T.IDGADGPIT.I	CID	26
2	O44359	Flageliform silk protein	Proteinase 10	290-319	771.960 (+3)	2312.858	2312.981	-0.1231	0	P.GGSGPGGVGPGGSGPGGVGPGGAGGPYGPG.G	CID + ETD	25
2	O44359	Flageliform silk protein	Proteinase 10	306-324	727.830 (+2)	1453.645	1453.658	-0.0132	0	G.GVGPGGAGGPYGPGGSGPG.G	CID	81
2	O44359	Flageliform silk protein	Proteinase 10	306-327	820.350 (+2)	1638.685	1638.738	-0.0533	0	G.GVGPGGAGGPYGPGGSGPGGAG.G	CID	48

2	O44359	Flageliform silk protein	Proteinase 10	306-328	848.850 (+2)	1695.685	1695.760	-0.0747	0	G.GVGPGGAGGPYGPGGSGPGGAGG.A	CID + ETD	49
2	O44359	Flageliform silk protein	Proteinase 10	306-333	1018.380 (+2)	2034.745	2034.914	-0.1690	0	G.GVGPGGAGGPYGPGGSGPGGAGGAGGPG.G	CID + ETD	44
2	O44359	Flageliform silk protein	Proteinase 10	306-334	1046.920 (+2)	2091.825	2091.935	-0.1105	0	G.GVGPGGAGGPYGPGGSGPGGAGGAGGPGG.A	CID	100
2	O44359	Flageliform silk protein	Proteinase 10	307-327	791.840 (+2)	1581.665	1581.717	-0.0518	0	G.VGPGGAGGPYGPGGSGPGGAG.G	CID	75
2	O44359	Flageliform silk protein	Proteinase 10	307-334	679.290 (+3)	2034.848	2034.914	-0.0663	0	G.VGPGGAGGPYGPGGSGPGGAGGAGGPGG.A	CID + ETD	40
2	O44359	Flageliform silk protein	Proteinase 10	311-320	789.340 (+1)	788.332	788.345	-0.0126	0	G.GAGGPYGPGG.S	CID	39
2	O44359	Flageliform silk protein	Proteinase 10	311-334	863.350 (+2)	1724.685	1724.750	-0.0649	0	G.GAGGPYGPGGSGPGGAGGAGGPGG.A	CID	94
2	O44359	Flageliform silk protein	Proteinase 10	337-345	748.250 (+1)	747.242	747.318	-0.0761	0	Y.GPGGSYGPG.G	CID	36
2	O44359	Flageliform silk protein	Proteinase 10	341-365	1001.890 (+2)	2001.765	2001.845	-0.0799	0	G.SYGPGGSGGPGGAGGPYGPGGEGPG.G	CID + ETD	80
2	O44359	Flageliform silk protein	Proteinase 10	342-351	805.310 (+1)	804.302	804.340	-0.0375	0	S.YGPGGSGGPG.G	CID	38
2	O44359	Flageliform silk protein	Proteinase 10	342-374	877.030 (+3)	2628.068	2628.126	-0.0585	0	S.YGPGGSGGPGGAGGPYGPGGEGPGGAGGPYGPG.G	CID + ETD	76
2	O44359	Flageliform silk protein	Proteinase 10	352-365	1129.450 (+1)	1128.442	1128.483	-0.0409	0	G.GAGGPYGPGGEGPG.G	CID	37
2	O44359	Flageliform silk protein	Proteinase 10	352-374	921.860 (+2)	1841.705	1841.796	-0.0915	0	G.GAGGPYGPGGEGPGGAGGPYGPG.G	CID + ETD	104
2	O44359	Flageliform silk protein	Proteinase 10	352-388	965.730 (+3)	2894.168	2894.264	-0.0964	0	G.GAGGPYGPGGEGPGGAGGPYGPGGAGGPYGPGGAGGP.Y	CID + ETD	106
2	O44359	Flageliform silk protein	Proteinase 10	366-388	892.870 (+2)	1783.725	1783.791	-0.0660	0	G.GAGGPYGPGGAGGPYGPGGAGGP.Y	CID	111
2	O44359	Flageliform silk protein	Proteinase 10	404-427	1016.400 (+2)	2030.785	2030.875	-0.0905	0	S.YGPGGAGGPYGPGGPYGPGGEGPG.G	CID	80

2	O44359	Flageliform silk protein	Proteinase 10	408-418	886.350 (+1)	885.342	885.398	-0.0554	0	G.GAGGPYGPGGP.Y	CID	23
2	O44359	Flageliform silk protein	Proteinase 10	408-427	829.340 (+2)	1656.665	1656.716	-0.0514	0	G.GAGGPYGPGGPYGPGGEGPG.G	CID	56
2	O44359	Flageliform silk protein	Proteinase 10	408-437	809.980 (+3)	2426.918	2427.051	-0.1335	0	G.GAGGPYGPGGPYGPGGEGPGGAGGPYGPGG.V	CID + ETD	69
2	O44359	Flageliform silk protein	Proteinase 10	410-437	767.290 (+3)	2298.848	2298.993	-0.1449	0	A.GGPYGPGGPYGPGGEGPGGAGGPYGPGG.V	CID + ETD	56
2	O44359	Flageliform silk protein	Proteinase 10	413-437	1044.890 (+2)	2087.765	2087.897	-0.1319	0	P.YGPGGPYGPGGEGPGGAGGPYGPGG.V	CID	52
2	O44359	Flageliform silk protein	Proteinase 10	552-577	964.360 (+2)	1926.705	1926.809	-0.1039	0	G.GSGPGGYGPGGSGPGGSGPGGSGPGG.Y	CID	42
2	O44359	Flageliform silk protein	Subtilisin	6-15	805.410 (+1)	804.402	804.340	0.0625	0	V.GPGGSGPGGY.G	CID	49
2	O44359	Flageliform silk protein	Subtilisin	273-290; 283-300	660.2815 (+2)	1318.5484	1318.6266	-0.0782	0	A.GPGGVGPGGSGPGGVGPG.G	CID + ETD	84
2	O44359	Flageliform silk protein	Subtilisin	276-290; 286-300	1108.3436 (+1)	1107.3363	1107.5309	-0.1946	0	G.GVGPGGSGPGGVGPG.G	CID	54
2	O44359	Flageliform silk protein	Subtilisin	281-290; 291-300; 301-310; 704-713	741.3361 (+1)	740.3288	740.3454	-0.0165	0	G.GSGPGGVGPG.G	CID	48
2	O44359	Flageliform silk protein	Subtilisin	281-300	732.2972 (+2)	1462.5798	1462.6801	-0.1003	0	G.GSGPGGVGPGGSGPGGVGPG.G	CID + ETD	90
2	O44359	Flageliform silk protein	Subtilisin	390-398	790.2277 (+1)	789.2204	789.3293	-0.1089	0	Y.GPGGEGGPY.G	CID	42

Table S2. The Modiro® characterization of flagelliform silk protein from the web silk produced by *N. clavipes*. Spot number, accession number, protein name, enzyme used, amino acid position, observed m/z, theoretical m/z, difference between observed m/z and theoretical m/z, number of missed cleavage sites, peptide sequences, PTMs, fragmentation method, Modiro® ion scores and significant scores are listed for all of the identified peptides.

Spot	Accession number	Protein	Enzyme	Amino acid position	m/z meas. [Da]	m/z theor. [Da]	Error [Da]	Z	Peptide sequence	PTMs / Fragmentation method	Modiro Ions Score	Modiro Score Sig.
1	O44358	Flagelliform silk protein	Glu-C	1-25	850.37	850.46	-0.0951	3	>.MGKGRHDTKAK$^{-50.28}$AKAMQVALASSIAE.L	Unknown shift / CID + ETD	310	89.6
1	O44358	Flagelliform silk protein	Glu-C	13 – 25	659.83	659.82	0.012	2	A.KAMQVALASSIAE.L	CID	220	100
1	O44358	Flagelliform silk protein	Glu-C	26 – 30	544.31	544.33	-0.0241	1	E.LVIAE.S	CID	255	99.8
1	O44358	Flagelliform silk protein	Glu-C	31 – 61	809.12	809.15	-0.0308	4	E.SSGGDVQRKTNVISNALRNALMSTTGSPNEE.F	ETD	396	99.8
1	O44358	Flagelliform silk protein	Glu-C	62 – 80	815.35	815.33	0.023	3	E.FVHEVQDLIQM^{Ox}LS$^{127.92}$QEQINE.V	Unknown shift / CID + ETD	273	82.6
1	O44358	Flagelliform silk protein	Glu-C	66 – 80	894.42	894.44	-0.0258	2	E.VQDLIQMLSQEQINE.V	CID	529	100
1	O44358	Flagelliform silk protein	Glu-C	77 – 109	906.64	906.61	0.0242	4	E.QINE$^{439.1}$VDTSGPGQYYRSSSSGGGGGGQGGPVVTE.T	Unknown shift / ETD	388	81.8
1	O44358	Flagelliform silk protein	Glu-C	81 – 109	900.7	900.74	-0.0426	3	E.VDTSGPGQYYRSSSSGGGGGGQGGPVVTE.T	CID + ETD	488	100
1	O44358	Flagelliform silk protein	Glu-C	504 – 521	1110.47	1110.44	0.0207	2	E.DLDITIDGA$^{346.04}$DGPITISEE.L	Unknown shift / CID	231	90.9
1	O44359	Flagelliform silk protein	Chymotrypsin	1-25	971.96	971.93	0.0224	2	>.GPGGVGPGGSGPGGYGPGGAGPGGY.G	CID	440	100
1	O44359	Flagelliform silk protein	Chymotrypsin	1-35	928.46	928.404	0.056	3	>.GPGGVGPGGSGPGGYGPG$^{54.17}$GAGPGGYGPGGSGPGGY.G	Unknown shift / CID + ETD	346	100
1	O44359	Flagelliform silk protein	Chymotrypsin	16 - 25; 459 – 468	405.75	405.67	0.0701	2	Y.GPGGAGP$^{21.14}$GGY.G	Unknown shift / CID	257	82.6
1	O44359	Flagelliform silk protein	Chymotrypsin	16 - 35; 459 – 478	754.41	754.39	0.0234	2	Y.GPGGAGPGGYGPGGS$^{-67.86}$GPGGY.G	Unknown shift / CID	226	89.8

1	O44359	Flagelliform silk protein	Chymotrypsin	26 - 45; 36 - 55; 46 - 65; 56 - 75; 469 - 488; 479 - 498; 489 - 508; 519 – 538	804.93	804.84	0.0878	2	Y.GPGGSGPGGYGPGGSGPGGY.G	CID	240	100
1	O44359	Flagelliform silk protein	Chymotrypsin	56 – 80	667.65	667.60	0.0507	3	Y.GPGGSGPGGYGPGG$^{-21.91}$SGPGGYGPGGY.G	Unknown shift / ETD	243	98.6
1	O44359	Flagelliform silk protein	Chymotrypsin	66 – 80	660.95	660.76	0.1824	2	Y.GPGGSGPG$^{84.36}$GYGPGGY.G	Unknown shift / CID	287	89.5
1	O44359	Flagelliform silk protein	Chymotrypsin	76 – 105	805.76	805.679	0.081	3	Y.GPGGYGPGGSGPGGYGPGGTGPGG$^{23.24}$SGPGGY.G	Unknown shift / CID + ETD	231	86.8
1	O44359	Flagelliform silk protein	Chymotrypsin	81 – 105	631.47	631.61	-0.1489	3	Y.GPGG$^{-68.44}$SGPGGYGPGGTGPGGSGPGGY.G	Unknown shift / CID + ETD	257	91.6
1	O44359	Flagelliform silk protein	Chymotrypsin	81 – 120	863.91	863.83	0.0743	4	Y.GPGGSGPGGY$^{350.3}$GPGGTGPGGSGPGGYGPGGSGPGGSGPGGY.G	Unknown shift / ETD	326	81.3
1	O44359	Flagelliform silk protein	Chymotrypsin	106 – 120	360.31	360.50	-0.1937	3	Y.GPGGSGP$^{-81.57}$GGSGPGGY.G	Unknown shift / CID + ETD	233	86.1
1	O44359	Flagelliform silk protein	Chymotrypsin	106 – 140	1105.62	1105.39	0.2281	3	Y.GPGGSGPGGSGPGGYGPGG$^{597.68}$SGPGGFGPGGSGPGGY.G	Unknown shift / CID + ETD	230	80
1	O44359	Flagelliform silk protein	Chymotrypsin	141 – 160	711.48	711.46	0.0236	2	Y.GPGGSGPGG$^{-73.73}$AGPGGVGPGGF.G	Unknown shift / CID	228	87.5
1	O44359	Flagelliform silk protein	Chymotrypsin	260 - 287; 683 – 710	1046.54	1046.50	0.0337	2	L.TISGAGGSGPGGAGPGGVGPGGSGPGGV$^{\sim L \sim}$.G	Substitution Val->Leu (V287, V710) / CID	224	87.5
1	O44359	Flagelliform silk protein	Chymotrypsin	317 – 336	751.88	751.81	0.0636	2	Y.GPGGSGPGGAGGAGGPGGAY^{Nitro}.G	Nitro (Y336) / CID	305	100
1	O44359	Flagelliform silk protein	Chymotrypsin	317 – 342	758.38	758.28	0.0908	3	Y.G$^{297.27}$PGGSGPGGAGGAGGPGGAYGPGGSY.G	Unknown shift / CID + ETD	250	86.7
1	O44359	Flagelliform silk protein	Chymotrypsin	337 - 342; 399 – 404	817.23	817.21	0.0226	1	Y.GP$^{280.}$GGSY.G	Unknown shift / CID	204	83.5
1	O44359	Flagelliform silk protein	Chymotrypsin	337 – 357	831.94	831.86	0.0792	2	Y.GPGGSYGPGGSGGPGGAGGPY.G	CID	399	100
1	O44359	Flagelliform silk protein	Chymotrypsin	337 – 371	937.81	937.734	0.076	3	Y.GPGGSYGPG$^{38.23}$GSGGPGGAGGPYGPGGEGPGGAGGPY.G	Unknown shift / CID + ETD	316	100

1	O44359	Flagelliform silk protein	Chymotrypsin	343 – 357	565.32	565.30	0.0214	2	Y.GPGGS$^{-14.86}$GGPGGAGGPY.G	Unknown shift / CID	476	100
1	O44359	Flagelliform silk protein	Chymotrypsin	343 – 380	791.1	791.07	0.0225	4	Y.GPGGSGGPGGAGGPYGPGGEGPGGAGG$^{193.09}$PYGPGGAGGPY.G	Unknown shift / ETD	308	85.7
1	O44359	Flagelliform silk protein	Chymotrypsin	358 - 371; 420 – 433	573.81	573.74	0.0609	2	Y.G$^{17.12}$PGGEGPGGAGGPY.G	Unknown shift / CID	278	99.1
1	O44359	Flagelliform silk protein	Chymotrypsin	358 – 380	930.46	930.40	0.0543	2	Y.GPGGEGPGGAGGP$^{17.11}$YGPGGAGGPY.G	Unknown shift / CID	318	100
1	O44359	Flagelliform silk protein	Chymotrypsin	372 - 380; 381 - 389; 405 - 413; 791 – 799	732.39	732.33	0.0589	1	Y.GPGGAGGPY.G	CID	204	100
1	O44359	Flagelliform silk protein	Chymotrypsin	372 – 389	731.45	731.32	0.1267	2	Y.GPGGAGGPYGPGGAGGPY.G	CID	241	96
1	O44359	Flagelliform silk protein	Chymotrypsin	381 – 398	752.4	752.32	0.0714	2	Y.GPGGAGGPYGPGGEGGPY.G	CID	402	100
1	O44359	Flagelliform silk protein	Chymotrypsin	390 – 413	1017.01	1016.93	0.0752	2	Y.GPGGEGGPYGPGGSY$^{11.15}$GPGGAGGPY.G	Unknown shift / CID	204	98.8
1	O44359	Flagelliform silk protein	Chymotrypsin	399 – 419	886.98	886.96	0.0212	2	Y.GPGGS$^{-5.82}$YGPGGAGGPYGPGGPY.G	Unknown shift / CID	404	100
1	O44359	Flagelliform silk protein	Chymotrypsin	405 – 433	791.1	791.017	0.0827	3	Y.GPGGAGGPYGPGGPYGPGGEGPGGAGGPY.G	ETD	325	100
1	O44359	Flagelliform silk protein	Chymotrypsin	414 – 433	837.93	837.86	0.0643	2	Y.G$^{17.13}$PGGPYGPGGEGPGGAGGPY.G	Unknown shift / CID	369	100
1	O44359	Flagelliform silk protein	Chymotrypsin	434 – 458	479.22	479.22	-0.0011	4	Y.GPGGVGPGGSGPGGYGP$^{-45.}$GGSGPGGY.G	Unknown shift / ETD	280	80.9
1	O44359	Flagelliform silk protein	Chymotrypsin	449 – 468	825.39	825.34	0.0452	2	Y.G$^{74.09}$PGGSGPGGYGPGGAGPGGY.G	Unknown shift / CID	278	85.9
1	O44359	Flagelliform silk protein	Chymotrypsin	449 – 478	868.22	868.00	0.2112	3	Y.GPGGSG$^{240.63}$PGGYGPGGAGPGGYGPGGSGPGGY.G	Unknown shift / CID + ETD	242	83.1
1	O44359	Flagelliform silk protein	Chymotrypsin	499 – 518	741.96	741.95	0.0137	2	Y.GPGGSGPGGYGSGGAG$^{-82.74}$PGGY.G	Unknown shift / CID	252	91.6
1	O44359	Flagelliform silk protein	Chymotrypsin	509 – 518	913.51	913.33	0.1781	1	Y.GSGGAGPGGY$^{134.18}$.G	Unknown shift / CID	224	98

1	O44359	Flagelliform silk protein	Chymotrypsin	509 – 528	523.67	523.55	0.1113	3	Y.GSG$^{3.33}$GAGPGGYGPGGSGPGGY.G	Unknown shift / CID + ETD	287	87.2
1	O44359	Flagelliform silk protein	Chymotrypsin	529 – 578	965.44	965.41	0.0284	4	Y.GPGGSGPGGYGPGGTGPGGTGPGGSGPGGYGPG GSGPGGSGPGGSGPGGY.G	CID + ETD	290	80.8
1	O44359	Flagelliform silk protein	Chymotrypsin	559 – 578	796.4	796.34	0.0578	2	Y.GPGGSGPGGS$^{\sim Y \sim}$GPGGSGPGGY.G	Substitution Ser->Tyr (S568) / CID	425	100
1	O44359	Flagelliform silk protein	Chymotrypsin	559 – 588	608.61	608.52	0.0881	4	Y.GPGGSGPGG$^{\sim R \sim}$SGPGGSGPGGYGPSGSGPGGY.G	Substitution Gly->Arg (G567) / ETD	380	93.7
1	O44359	Flagelliform silk protein	Chymotrypsin	579 – 598	549.25	549.23	0.0211	3	Y.GPSGSGPGG$^{-5.95}$YGPSGSGPGGY.G	Unknown shift / CID	263	96.4
1	O44359	Flagelliform silk protein	Chymotrypsin	746 – 769	816.43	816.28	0.1464	3	Y.GPGGSGSGGVGPGG$^{505.44}$YGPGGSGGFY.G	Unknown shift / CID + ETD	355	98.6
1	O44359	Flagelliform silk protein	Chymotrypsin	770 – 784	685.08	685.29	-0.2166	2	Y.GPGGSEGPYGP$^{-13.42}$SGTY.G	Unknown shift / CID	206	80.6
1	O44359	Flagelliform silk protein	Chymotrypsin	785 – 799	584.04	584.25	-0.2198	2	Y.GSGGGYGPGGAGG$^{-43.43}$PY.G	Unknown shift / CID	204	83.3
1	O44359	Flagelliform silk protein	Chymotrypsin	840 – 848	617.84	617.74	0.0986	2	F.NYQ$^{101.2}$MFGNML.S	Unknown shift / CID	291	84.9
1	O44359	Flagelliform silk protein	Chymotrypsin	849 – 866	610.71	610.68	0.0325	3	L.SQYSSGSGTCNP$^{-10.69}$NNVNVL.M	Unknown shift / CID + ETD	322	87.7
1	O44359	Flagelliform silk protein	Chymotrypsin	852 – 870	674.07	673.95	0.1159	3	Y.SSG$^{127.35}$SGTCNPNNVNVLMDAL.L	Unknown shift / CID	206	87.9
1	O44359	Flagelliform silk protein	Chymotrypsin	875 – 897	858.15	858.00	0.1428	3	L.HCLSNHGSSSFAPSPT$^{236.43}$PAAM^{Ox}SAY.S	Unknown shift / CID + ETD	335	100
1	O44359	Flagelliform silk protein	Chymotrypsin	878 – 885	412.75	412.67	0.0775	2	L.S$^{2.15}$NHGSSSF.A	Unknown shift / CID	239	99.9
1	O44359	Flagelliform silk protein	Chymotrypsin	886 – 907	794.84	794.68	0.1561	3	F.APSP$^{74.47}$TPAAM^{Ox}SAYSNSVGRM^{Ox}FAY.-	Unknown shift / CID + ETD	237	80.8
2	O44358	Flagelliform silk protein	Glu-C	1-25	1363.2	1363.19	0.006	2	>.MGKGRHDTKAKAKAMQV$^{126.01}$ALASSIAE.L	Unknown shift / CID + ETD	259	85.2

2	O44358	Flagelliform silk protein	Glu-C	13 – 25	659.83	659.82	0.012	2	A.KAMQVALASSIAE.L	CID + ETD	228	99.8
2	O44358	Flagelliform silk protein	Glu-C	26 – 30	544.33	544.33	-0.0041	1	E.LVIAE.S	ETD	243	99.9
2	O44358	Flagelliform silk protein	Glu-C	31 – 61	1083.83	1083.86	-0.0337	3	E.SSGGDVQRKTNVISNALRNALM^{Ox}STTGSPNEE.F	Oxidation (M52) / CID + ETD	317	100
2	O44358	Flagelliform silk protein	Glu-C	31 – 61	809.35	809.39	-0.0468	4	E.SSGGDVQR^{Deamid}KTNVISNALRNALMSTTGSPNEE.F	Deamidation (R38) / ETD	452	98.8
2	O44358	Flagelliform silk protein	Glu-C	62 – 80	757.07	757.05	0.021	3	E.FVHEVQDLIQML$^{-30.92}$SQEQINE.V	Unknown shift / CID + ETD	299	93.1
2	O44358	Flagelliform silk protein	Glu-C	66 – 80	894.51	894.44	0.0642	2	E.VQDLIQMLSQEQINE.V	CID	525	100
2	O44358	Flagelliform silk protein	Glu-C	77 – 109	1078.52	1078.48	0.0347	3	E.QINEVDTSGPGQYYRSSSSGGGGGGQGG$^{49.1}$PVVTE .T	Unknown shift / CID + ETD	205	91.8
2	O44358	Flagelliform silk protein	Glu-C	81 – 109	900.73	900.74	-0.0126	3	E.VDTSGPGQYYRSSSSGGGGGGQGGPVVTE.T	CID + ETD	523	100
2	O44358	Flagelliform silk protein	Glu-C	504 – 520	944.93	944.928	0.002	2	E.DLDITIDGADGPITI$^{144.}$SE.E	Unknown shift / CID	234	87.5
2	O44359	Flagelliform silk protein	Chymotrypsin	1 - 15; 434 – 448	594.76	594.76	0	2	>.GPGGVGPGGSGPGGY.G	CID	277	100
2	O44359	Flagelliform silk protein	Chymotrypsin	1 – 25	979.94	979.935	0.0078	2	>.GPGGVGPGGSGPGGYGPGGAGPGGY.G	CID	243	100
2	O44359	Flagelliform silk protein	Chymotrypsin	1 – 35	928.71	928.70	0.0172	3	>.GPGGVGPGGSGPGGYGPGG$^{54.92}$AGPGGYGPGGSG PGGY.G	Unknown shift / CID	210	100
2	O44359	Flagelliform silk protein	Chymotrypsin	1 – 45	1172.51	1172.51	0.0016	3	>.GPGGVGPGGSGPGGYGPGGAGPGGYGPGGSGPG GYGPGGSGPGGY.G	CID + ETD	251	100
2	O44359	Flagelliform silk protein	Chymotrypsin	16 - 45; 459 – 488	1189.50	1189.50	0.0009	2	Y.GPGGAGPGGYGPGGSGPGGYGPGGSGPGGY.G	CID	290	100
2	O44359	Flagelliform silk protein	Chymotrypsin	16 - 45; 459 – 488	1197.49	1197.50	-0.0095	2	Y.GPGGAGPGGYGPGGSGPGGYGPGGSGPGGY.G	CID	207	100

2	O44359	Flagelliform silk protein	Chymotrypsin	26 - 35; 36 - 45; 46 - 55; 56 - 65; 66 - 75; 81 - 90; 449 - 458; 469 - 478; 479 - 488; 489 - 498; 499 - 508; 519 - 528; 529 - 538; 599 – 608	403.17	403.17	0.0003	2	Y.GPGGSGPGGY.G	CID	254	100
2	O44359	Flagelliform silk protein	Chymotrypsin	26 - 45; 36 - 55; 46 - 65; 56 - 75; 469 - 488; 479 - 498; 489 - 508; 519 – 538	812.35	812.35	0.001	2	Y.GP^{Ox}GGSGPGGYGP^{Ox}GGSGPGGY.G	Hydroxylation (P27, P37, P47, P57, P67, P470, P480, P490, P500, P520, P530) / CID	230	100
2	O44359	Flagelliform silk protein	Chymotrypsin	56 – 80	973.92	973.91	0.0063	2	Y.GPGGSGPGGYGPGGSGPGGYGPGGY.G	CID	201	99.9
2	O44359	Flagelliform silk protein	Chymotrypsin	66 – 80	610.76	610.77	-0.0005	2	Y.GPGGSGPGGYGPGGY.G	CID	308	100
2	O44359	Flagelliform silk protein	Chymotrypsin	66 – 90	974.41	974.43	-0.0133	2	Y.GPGGSGPGGYGP$^{-75.02}$GGYGPGGSGPGGY.G	Unknown shift / CID	210	100
2	O44359	Flagelliform silk protein	Chymotrypsin	76 – 90	586.76	586.74	0.0249	2	Y.GPGGY$^{-63.99}$GPGGSGPGGY.G	Unknown shift / CID	344	100
2	O44359	Flagelliform silk protein	Chymotrypsin	81 – 105	981.91	981.89	0.0248	2	Y.GPGGSGPGGYGPG$^{1.98}$GTGPGGSGPGGY.G	Unknown shift / CID + ETD	221	100
2	O44359	Flagelliform silk protein	Chymotrypsin	81 – 120	1030.77	1030.77	-0.0027	3	Y.GPGGSGPGGYGPGGT^{-12}GPGGSGPGGYGPGGSGPGGSGPGGY.G	Unknown shift / CID + ETD	205	84.2
2	O44359	Flagelliform silk protein	Chymotrypsin	91 – 105	618.76	618.75	0.0045	2	Y.GPGGT$^{62.01}$GPGGSGPGGY.G	Unknown shift / CID	202	100
2	O44359	Flagelliform silk protein	Chymotrypsin	91 – 120	558.52	558.48	0.0423	4	Y.GPGGTGPGGSGP$^{-84.92}$GGYGPGGSGPGGSGPGGY.G	Unknown shift / ETD	253	86.9
2	O44359	Flagelliform silk protein	Chymotrypsin	106 – 120	580.75	580.752	-0.0013	2	Y.GPGGSGPGGSGPGGY.G	CID	274	100

2	O44359	Flagelliform silk protein	Chymotrypsin	106 – 135	1181.50	1181.50	-0.0022	2	Y.GPGGSGPGGSGPGGYGPGGSGPGGFGPGGS$^{\sim Y \sim}$.G	Substitution Ser->Tyr (S135) / CID	329	100
2	O44359	Flagelliform silk protein	Chymotrypsin	106 – 140	1050.45	1050.39	0.062	3	Y.GPGGSGPGGSGPGGYGPGGSGPGGFGPGGS$^{432.18}$GPGGY.G	Unknown shift / CID + ETD	239	99.6
2	O44359	Flagelliform silk protein	Chymotrypsin	141 – 160	709.83	709.84	-0.019	2	Y.GPGGS$^{-77.03}$GPGGAGPGGVGPGGF.G	Unknown shift / CID	218	91
2	O44359	Flagelliform silk protein	Chymotrypsin	243 - 259; 666 – 682	830.42	830.43	-0.0118	2	L.DITID$^{-99.01}$GADGPITISEEL.T	Unknown shift / CID	210	100
2	O44359	Flagelliform silk protein	Chymotrypsin	317 – 336	743.33	743.33	-0.0001	2	Y.GPGGSGPGGAGGAGGPGGAY.G	/ CID + ETD	414	100
2	O44359	Flagelliform silk protein	Chymotrypsin	317 – 342	861.71	861.62	0.0906	3	Y.GPGGSGPGGAGGAGGPGGAYGPGGSY$^{607.27}$.G	Unknown shift / CID + ETD	217	99.9
2	O44359	Flagelliform silk protein	Chymotrypsin	337 – 357	839.85	839.85	0.0013	2	Y.GPGGSYGPGGSGGPGGAGGPY.G	CID	201	100
2	O44359	Flagelliform silk protein	Chymotrypsin	337 – 371	920.07	920.04	0.0329	3	Y.GPGGSYGPGG$^{-14.98}$SGGPGGAGGPYGPGGEGPGGAGGPY.G	Unknown shift / CID + ETD	233	100
2	O44359	Flagelliform silk protein	Chymotrypsin	343 – 357	580.75	580.752	-0.0015	2	Y.GPGGSGGPGGAGGPY.G	CID	314	100
2	O44359	Flagelliform silk protein	Chymotrypsin	343 – 371	757.66	757.66	-0.0013	3	Y.GPGGSGGPGGAGGPYGPGGEGPGGAGGPY.G	CID	257	100
2	O44359	Flagelliform silk protein	Chymotrypsin	343 – 380	995.43	995.43	0.0036	3	Y.GPGGSGGPGGAGGPYGPGGEGPGGAGGPYGPGGAGGPY.G	CID	211	89.2
2	O44359	Flagelliform silk protein	Chymotrypsin	358 - 371; 420 – 433	566.32	566.24	0.0789	2	Y.GPGGEGPGGA$^{2.16}$GGPY.G	Unknown shift / CID	217	99.1
2	O44359	Flagelliform silk protein	Chymotrypsin	358 – 380	928.907	928.91	-0.0066	2	Y.GPGGE^{Me}GPGGAGGPYGPGGAGGPY.G	Methylation (E362) / CID	231	100
2	O44359	Flagelliform silk protein	Chymotrypsin	358 – 380	929.90	929.90	0.0009	2	Y.GPGGEGPGGAGGPYGPGGAGGPY.G	CID	232	100
2	O44359	Flagelliform silk protein	Chymotrypsin	358 – 389	714.78	714.78	0.0039	4	Y.G$^{300.01}$PGGEGPGGAGGPYGPGGAGGPYGPGGAGGPY.G	Unknown shift / ETD	255	95.5
2	O44359	Flagelliform silk protein	Chymotrypsin	372 - 380; 381 - 389; 405 - 413; 791 – 799	366.66	366.66	0.0004	2	Y.GPGGAGGPY.G	ETD	230	99.9

2	O44359	Flagelliform silk protein	Chymotrypsin	372 – 389	731.32	731.32	-0.0007	2	Y.GPGGAGGPYGPGGAGGPY.G	CID	263	100
2	O44359	Flagelliform silk protein	Chymotrypsin	381 – 398	760.32	760.326	-0.0025	2	Y.GPGGAGGPYGPGGEGGPY.G	CID	233	100
2	O44359	Flagelliform silk protein	Chymotrypsin	390 – 404	651.79	651.76	0.0328	2	Y.GPGGEGGPYGPGGSY$^{-5.97}$.G	Unknown shift / CID	299	100
2	O44359	Flagelliform silk protein	Chymotrypsin	399 – 413	631.302	631.27	0.0265	2	Y.G$^{11.05}$PGGSYGPGGAGGPY.G	Unknown shift / CID	203	100
2	O44359	Flagelliform silk protein	Chymotrypsin	399 – 419	894.89	894.90	-0.0029	2	Y.GPGGS$^{\sim P\sim}$YGPGGAGGPYGPGGPY.G	Substitution Ser->Pro (S403) / CID	225	100
2	O44359	Flagelliform silk protein	Chymotrypsin	405 – 419	638.78	638.78	0.0014	2	Y.GPGGAGGPYGPGGPY.G	CID	297	100
2	O44359	Flagelliform silk protein	Chymotrypsin	405 – 419	638.80	638.78	0.0258	2	Y.GPGGAGGPYGP^{Ox}GGPY.G	Hydroxylation (P415) / CID	203	97.1
2	O44359	Flagelliform silk protein	Chymotrypsin	414 – 433	837.36	837.36	0.0005	2	Y.GPGGPYGPGGEGPGGAGGPY.G	CID	284	100
2	O44359	Flagelliform silk protein	Chymotrypsin	420 – 448	1142.00	1142.00	0.0088	2	Y.GPGGEGPGGAGGPYGPGGVGPGGSGPGGY.G	CID + ETD	214	100
2	O44359	Flagelliform silk protein	Chymotrypsin	420 – 458	1018.78	1018.76	0.0213	3	Y.GPGGEGPGGAGGPYGPGGVGPGGSGPGGYGPGG$^{-14.98}$SGPGGY.G	Unknown shift / CID + ETD	216	99.9
2	O44359	Flagelliform silk protein	Chymotrypsin	434 – 458	667.628	667.62	-0.0013	3	Y.GPGGVGPGGSGPGGYGPGGS^{Ac}GPGGY.G	Acetylation (S453) / CID	205	100
2	O44359	Flagelliform silk protein	Chymotrypsin	434 – 468	906.39	906.404	-0.0104	3	Y.GPGGV$^{-12.02}$GPGGSGPGGYGPGGSGPGGYGPGGAGPGGY.G	Unknown shift / CID + ETD	207	99.5
2	O44359	Flagelliform silk protein	Chymotrypsin	449 – 478	793.674	793.672	0.002	3	Y.GPGGSGPGGYGPGGA$^{16.99}$GPGGYGPGGSGPGGY.G	Unknown shift / CID + ETD	209	99.9
2	O44359	Flagelliform silk protein	Chymotrypsin	499 – 518	604.95	604.892	0.0653	3	Y.GPGGSGPGGYGSGGAGPGG$^{247.2}$Y.G	Unknown shift / CID + ETD	224	96.5
2	O44359	Flagelliform silk protein	Chymotrypsin	499 – 528	811.31	811.324	-0.0132	3	Y.GPGGSGPGGYGSGGAGPGGYGPGGSGPGGY.G	CID	202	84.1
2	O44359	Flagelliform silk protein	Chymotrypsin	519 – 558	1055.78	1055.78	0.0054	3	Y.GPGGSGPGGYGPGGSGPGGYGPGGTGPG$^{49.01}$GTGPGGSGPGGY.G	Unknown shift / CID + ETD	210	98.7

2	O44359	Flagelliform silk protein	Chymotrypsin	559 – 578	532.22	532.22	0.0056	3	Y.GPGGSGPGGSGPGGSGPGG$^{79.02}$Y.G	Unknown shift / CID + ETD	201	90.8
2	O44359	Flagelliform silk protein	Chymotrypsin	559 – 588	817.35	817.33	0.0252	3	Y.GPGGSGPGGSGP$^{118.07}$GGSGPGGYGPSGSGPGGY.G	Unknown shift / CID + ETD	204	98.9
2	O44359	Flagelliform silk protein	Chymotrypsin	579 - 588; 589 – 598	449.69	449.68	0.0145	2	Y.GPSGS$^{63.03}$GPGGY.G	Unknown shift / CID	201	89.4
2	O44359	Flagelliform silk protein	Chymotrypsin	589 – 608	804.34	804.34	-0.0075	2	Y.G$^{-14.01}$PSGSGPGGYGPGGSGPGGY.G	Unknown shift / CID	243	100
2	O44359	Flagelliform silk protein	Chymotrypsin	746 – 768	889.89	889.89	0.0012	2	Y.GPGGSGSGGVGPGGYGPGGSGGF.Y	CID	211	100
2	O44359	Flagelliform silk protein	Chymotrypsin	746 – 769	971.42	971.42	-0.0016	2	Y.GPGGSGSGGVGPGGYGPGGSGGFY.G	CID	201	100
2	O44359	Flagelliform silk protein	Chymotrypsin	769 – 778	482.74	482.72	0.0214	2	F.YGP$^{-18.93}$GGSEGPY.G	Unknown shift / CID + ETD	204	86.3
2	O44359	Flagelliform silk protein	Chymotrypsin	770 – 778	533.26	533.17	0.0919	2	Y.GPGGSEGP$^{245.18}$Y.G	Unknown shift / CID	204	96.5
2	O44359	Flagelliform silk protein	Chymotrypsin	770 – 784	691.796	691.79	-0.0006	2	Y.GPGGSEGPYGPSGTY.G	CID	208	100
2	O44359	Flagelliform silk protein	Chymotrypsin	779 – 790	642.79	642.72	0.071	2	Y.G$^{225.14}$PSGTYGSGGGY.G	Unknown shift / CID	211	99.5
2	O44359	Flagelliform silk protein	Chymotrypsin	785 – 799	620.76	620.76	-0.0006	2	Y.GSGGGYGPGG$^{\sim S\sim}$AGGPY.G	Substitution Gly->Ser (G794) / CID	203	99.9
2	O44359	Flagelliform silk protein	Chymotrypsin	785 – 808	635.62	635.61	0.0148	3	Y.GSGGGYGPGGAGGPY$^{\sim N\sim}$GPGSPGGAY.G	Substitution Tyr->Asn (Y799) / CID + ETD	212	96.5
2	O44359	Flagelliform silk protein	Chymotrypsin	800 – 817	712.80	712.83	-0.0291	2	Y.GPGSPGGAYGPGSP$^{-81.05}$GGAY.Y	Unknown shift / CID	209	100
2	O44359	Flagelliform silk protein	Chymotrypsin	818 – 839	794.01	794.01	-0.0027	3	Y.YPSSRVPDM^{Ox}VN^{Deamid}GIM^{Ox}SAM^{Ox}QGSGF.N	Deamidation (N828); Oxidation (M826, M831, M834) / CID	208	80.9

2	O44359	Flagelliform silk protein	Chymotrypsin	818 – 841	860.40	860.36	0.0432	3	Y.YPSSRVPDM$^{-28.95}$VNGIMSAMQGSGFNY.Q	Unknown shift / CID + ETD	211	85.2
2	O44359	Flagelliform silk protein	Chymotrypsin	842 – 848	421.77	421.69	0.0857	2	Y.QMF$^{2.17}$GNML.S	Unknown shift / CID	206	80
2	O44359	Flagelliform silk protein	Chymotrypsin	849 – 870	1113.99	1114.01	-0.0189	2	L.SQYSSG$^{-117.03}$SGTC^{CAMe}NPNNVNVLM^{Ox}DAL.L	Unknown shift / CID	213	94.7
2	O44359	Flagelliform silk protein	Chymotrypsin	852 – 871	1032.48	1032.47	0.0137	2	Y.SSGSGTC^{CAMe}NPNNVN^{-D-}VLMDALL.A	Substitution Asn->Asp (D864) / CID	205	82.9
2	O44359	Flagelliform silk protein	Chymotrypsin	878 – 885	412.72	412.67	0.0568	2	L.SNHGSS$^{2.11}$SF.A	Unknown shift / CID	419	97.4
2	O44359	Flagelliform silk protein	Chymotrypsin	898 – 905	527.27	527.216	0.0557	2	Y.S$^{156.11}$NSVGRMF.A	Unknown shift / CID	204	94.4

Table S3. Proteolytic fragments of flagelliform silk protein from the web silk produced by *N. clavipes*, obtained by digestions with trypsin, chymotrypsin, Glu-C/V8 protease, subtilisin and proteinase 10. The peptides are presented according to the protein domain to which they belong to (N-terminal domain, central repetitive domain and C-terminal domain), as well are presented the amino acid position, peptide sequence, the m/z value of the respective molecular ion, charge state, spectral counting during mass spetrometric analysis and normalized value of spectral counting. (*) Sequences showing residues with PTMs (assigned in red).

Amino acid position	Peptide sequence	m/z (charge)	Spectral counting	Normalized spectral counting (integer number)
	**Non-repetitive N-terminal domain**			
1-25	MGKGRHDTKAKAKAMQVALASSIAE	850.37 (+3)	6	1.2 (1)
13-25	KAMQVALASSIAE	659.83 (+2)	5	1.0 (1)
14-38	AMQVALASSIAELVIAESSGGDVQR	834.72 (+3)	5	1.0 (1)
26-30	LVIAE	544.31 (+1)	7	1.4 (1)
31-60	SSGGDVQRKTNVISNALRNALMSTTGSPNE	1078.49 (+3)	8	1.6 (2)
31-61	SSGGDVQRKTNVISNALRNALMSTTGSPNEE	809.39 (+4)	8	1.6 (2)
31-65	SSGGDVQRKTNVISNALRNALMSTTGSPNEEFVHE	941.41 (+4)	5	1.0 (1)

39-48	KTNVISNALR	558.31 (+3)	6	1.2 (1)
40-48	TNVISNALR	494.23 (+2)	8	1.6 (2)
62-80	FVHEVQDLIQMLSQEQINE	757.07 (+3)	6	1.2 (1)
66-80	VQDLIQMLSQEQINE	894.42 (+2)	7	1.4 (1)
77-109	QINEVDTSGPGQYYRSSSSGGGGGGQGGPVVTE	906.64 (+4)	5	1.0 (1)
81-109	VDTSGPGQYYRSSSSGGGGGGQGGPVVTE	900.73 (+3)	5	1.0 (1)
	**Central repetitive domain composed of 3 modules**			
110-124; 543-557; 845-859; 1278-1292; 1580-1594; 2013-2027	GPGGVGPGGSGPGGY	594.76 (+2)	30	6.0 (6)
110-134; 845-869; 1580-1604	GPGGVGPGGSGPGGYGPGGAGPGGY	979.94 (+2)	15	3.0 (3)
110-144; 845-879; 1580-1614	GPGGVGPGGSGPGGYGPGGAGPGGYGPGGSGPGGY	928.71 (+3)	15	3.0 (3)
110-154; 845-889; 1580-1624	GPGGVGPGGSGPGGYGPGGAGPGGYGPGGSGPGGYGPGGSGPGGY	1172.51 (+3)	15	3.0 (3)
113-123; 405-415; 546-556; 848-858; 1140-1150; 1281-1291; 1583-1593; 1875-1885; 2016-2026	GVGPGGSGPGG	798.34 (+1)	45	9.0 (9)
114-123; 386-395; 406-415; 547-556; 809-818; 849-858; 1121-1130; 1141-1150; 1282-1291; 1544-1553; 1584-1593; 1856-1865; 1876-1885; 2017-2026; 2279-2288	VGPGGSGPGG	741.34 (+1)	75	15.0 (15)
114-127; 547-560; 849-862; 1282-1295; 1584-1597; 2017-2030	VGPGGSGPGGYGPG	1115.41 (+1)	30	6.0 (6)
114-128; 547-561; 849-863; 1282-1294; 1584-1598; 2017-2031	VGPGGSGPGGYGPGG	586.76 (+2)	30	6.0 (6)
114-133; 849-868; 1584-1603	VGPGGSGPGGYGPGGAGPGG	756.33 (+2)	15	3.0 (3)
114-137; 849-872; 1584-1607	VGPGGSGPGGYGPGGAGPGGYGPG	943.41 (+2)	15	3.0 (3)

115-124; 135-144; 145-154; 155-164; 165-174; 180-189; 190-199; 205-214; 220-229; 548-557; 558-567; 578-587; 588-597; 598-607; 608-617; 628-637; 638-647; 658-667; 678-687; 708-717; 850-859; 870-879; 880-889; 890-899; 900-909; 915-924; 925-934; 940-949; 955-964; 1283-1292; 1293-1302; 1313-1322; 1323-1332; 1333-1342; 1343-1352; 1363-1372; 1373-1382; 1393-1402; 1413-1422; 1443-1452; 1585-1594; 1605-1614; 1615-1624; 1625-1634; 1635-1644; 1650-1659; 1660-1669; 1675-1684; 1690-1699; 2018-2027; 2028-2037; 2048-2057; 2058-2067; 2068-2077; 2078-2087; 2098-2107; 2108-2117; 2128-2137; 2148-2157; 2178-2187	GPGGSGPGGY	805.30 (+1)	300	60.0 (60)
124-143; 567-586; 859-878; 1302-1321; 1594-1613; 2037-2056	YGPGGAGPGGYGPGGSGPGG	788.32 (+2)	30	6.0 (6)
125-134; 568-577; 860-869; 1303-1312; 1595-1604; 2038-2047	GPGGAGPGGY	405.75 (+2)	30	6.0 (6)
125-144; 568-587; 860-879; 1303-1322; 1595-1614; 2038-2057	GPGGAGPGGYGPGGSGPGGY	788.34 (+2)	30	6.0 (6)
125-154; 568-597; 860-889; 1303-1332; 1595-1624; 2038-2067	GPGGAGPGGYGPGGSGPGGYGPGGSGPGGY	1189.50 (+2)	30	6.0 (6)
129-147;572-590; 622-640; 864-882; 1307-1325; 1357-1375; 1599-1617; 2042-2060; 2092-2110	AGPGGYGPGGSGPGGYGPG	759.79 (+2)	45	9.0 (9)
129-163; 572-606; 864-898; 1307-1341; 1599-1633; 2042-2076	AGPGGYGPGGSGPGGYGPGGSGPGGYGPGGSGPGG	906.36 (+3)	30	6.0 (6)
133-153; 163-183; 576-596; 626-646; 868-888; 898-918; 1311-1331; 1361-1381; 1603-1623; 1633-1653; 2046-2066; 2096-2116	GYGPGGSGPGGYGPGGSGPGG	824.82 (+2)	60	12.0 (12)
133-157; 576-600; 626-650; 868-892; 1311-1335; 1361-1385; 1603-1627; 2046-2070; 2096-2120	GYGPGGSGPGGYGPGGSGPGGYGPG	1011.88 (+2)	45	9.0 (9)
133-163; 576-606; 868-898; 1311-1341; 1603-1633; 2046-2076	GYGPGGSGPGGYGPGGSGPGGYGPGGSGPGG	812.32 (+3)	30	6.0 (6)

133-167; 576-610; 868-902; 1311-1345; 1603-1637; 2046-2080	GYGPGGSGPGGYGPGGSGPGGYGPGGSGPGGYGPG	937.08 (+3)	30	6.0 (6)
134-147; 154-167; 189-202; 557-570; 577-590; 597-610; 627-640; 707-720; 869-882; 889-902; 924-937; 1292-1305; 1312-1325; 1332-1345; 1362-1375; 1442-1455; 1604-1617; 1624-1637; 1659-1672; 2027- 2040; 2047-2060; 2067- 2080; 2097-2110; 2177-2190	YGPGGSGPGGYGPG	590.22 (+2)	120	24.0 (24)
134-152; 154-172; 577-595; 597-615; 627-645; 869-907; 1312-1330; 1332-1350; 1362-1380; 1604- 1622; 1624-1642; 2047-2065; 2067-2085; 2097-2115	YGPGGSGPGGYGPGGSGPG	767.81 (+2)	70	14.0 (14)
134-173; 577-616; 627-646; 869-908; 1312-1351; 1362-1381; 1604-1643; 2047-2086; 2097-2116	YGPGGSGPGGYGPGGSGPGG	796.30 (+2)	45	9.0 (9)
134-157; 577-600; 627-650; 809-892; 1312-1335; 1362-1385; 1604-1627; 2047-2070; 2097-2120	YGPGGSGPGGYGPGGSGPGGYGPG	983.38 (+2)	45	9.0 (9)
134-158; 577-601; 627-651; 809-893; 1312-1336; 1362-1386; 1604-1628; 2047-2071; 2097-2121	YGPGGSGPGGYGPGGSGPGGYGPGG	1011.89 (+2)	45	9.0 (9)
134-162; 577-605; 869-897; 1312-1340; 1604-1632; 2047-2075	YGPGGSGPGGYGPGGSGPGGYGPGGSGPG	774.28 (+3)	30	6.0 (6)
134-163; 577-615; 869-898; 1312-1341; 1604-1633; 2047-2076	YGPGGSGPGGYGPGGSGPGGYGPGGSGPGG	793.31 (+3)	30	6.0 (6)
134-172; 577-624; 869-907; 1312-1350; 1604-1642; 2047-2085	YGPGGSGPGGYGPGGSGPGGYGPGGSGPGGYGPGGSGPG	1036.38 (+3)	30	6.0 (6)
135-144, 145-154, 155-164, 165-174, 175-184, 190-199, 558-567, 578-587, 588-597, 598-607, 608-617, 628-637, 638-647, 708-617, 870-879, 880-889, 890-899, 900-909, 910-919, 925-934, 1293-1302, 1313-1322, 1323-1332, 1333-1342, 1343-1352, 1363-1372, 1373-1382, 1443-1452, 1605-1614, 1615-1624, 1625-1634, 1635-1644, 1645-1654, 1660-1669, 2028-2037, 2048-2057, 2058-2067, 2068-2077, 2078-2087, 2098-2107, 2108-2117, 2178-2187	GPGGSGPGGY (*) hydroxyproline	411.210 (+2)	210	42.0 (42)

135-154; 155-174; 180-199; 548-567; 578-597; 598-617; 628-647; 870-889; 890-909; 915-934; 1283-1302; 1313-1332; 1333-1352; 1363-1382; 1605-1624; 1625-1644; 1650-1669; 2018-2037; 2048-2067; 2068-2087; 2098-2117	GPGGSGPGGYGPGGSGPGGY (*) hydroxyproline	812.35 (+2)	105	21.0 (21)
138-153; 158-173; 183-198; 208-223; 551-566; 581-596; 601-616; 631-646; 701-716; 873-888; 893-908; 918-933; 943-958; 1286-1301; 1316-1331; 1336-1351; 1366-1381; 1436-1469; 1608-1623; 1628-1643; 1653-1668; 1678-1693; 2021-2036; 2051-2066; 2071-2086; 2101-2116; 2171-2186	GSGPGGYGPGGSGPGG	609.23 (+2)	135	27.0 (27)
138-162; 581-605; 873-897; 1316-1340; 1608-1632; 2051-2075	GSGPGGYGPGGSGPGGYGPGGSGPG	973.87 (+2)	30	6.0 (6)
138-167; 581-610; 873-902; 1316-1345; 1608-1637; 2051-2081	GSGPGGYGPGGSGPGGYGPGGSGPGGYGPG	793.34 (+3)	30	6.0 (6)
138-172; 581-615; 873-907; 1316-1350; 1608-1642; 2051-2085	GSGPGGYGPGGSGPGGYGPGGSGPGGYGPGGSGPG	911.69 (+3)	30	6.0 (6)
138-173; 581-616; 873-908; 1316-1351; 1608-1643; 2051-2086	GSGPGGYGPGGSGPGGYGPGGSGPGGYGPGGSGPGG	930.69 (+3)	30	6.0 (6)
139-173; 582-616; 874-908; 1317-1351; 1609-1643; 2052-2086	SGPGGYGPGGSGPGGYGPGGSGPGGYGPGGSGPGG	911.69 (+3)	30	6.0 (6)
164-197; 899-932; 1634-1667	YGPGGSGPGGYGPGGSGPGGYGPGGYGPGGSGPG	944.70 (+3)	15	3.0 (3)
134-158; 445-469; 577-601; 627-651; 869-893; 1312-1336; 1362-1386; 1604-1628; 2097-2121	GPGGSGPGGYGPGGSGPGGYGPGGY	667.65 (+3)	50	10.0 (10)
144-158; 455-469; 587-601; 637-651; 879-893; 1322-1336; 1372-1386; 1614-1628; 2107-2121	GPGGSGPGGYGPGGY	610.76 (+2)	45	9.0 (9)
144-168; 455-479; 587-611; 637-661; 879-903; 1322-1346; 1372-1396; 1614-1638; 2107-2131	GPGGSGPGGYGPGGYGPGGSGPGGY	974.41 (+2)	45	9.0 (9)
185-214; 920-929; 1655-1394	GPGGYGPGGSGPGGYGPGGTGPGGSGPGGY	805.76 (+3)	15	3.0 (3)

130-144; 150-164; 185-199; 533-547; 573-587; 593-607; 623-637; 703-717; 865-879; 885-899; 920-934; 1288-1302; 1308-1322; 1328-1342; 1358-1372; 1438-1452; 1600-1614; 1620-1634; 1655-1669; 2023-2037; 2043-2057; 2063-2077; 2093-2107; 2173-2187	GPGGYGPGGSGPGGY	618.76 (+2)	120	24.0 (24)
190-214; 925-949; 1660-1684	GPGGSGPGGYGPGGTGPGGSGPGGY	631.47 (+3)	15	3.0 (3)
190-229; 925-964; 1660-1699	GPGGSGPGGYGPGGTGPGGSGPGGYGPGGSGPGGSGPGGY	863.91 (+4)	15	3.0 (3)
200-214; 653-667; 935-949; 1388-1402; 1670-1684; 2123-2137	GPGGTGPGGSGPGGY	618.76 (+2)	30	6.0 (6)
200-229; 935-964; 1670-1699	GPGGTGPGGSGPGGYGPGGSGPGGSGPGGY	558.52 (+4)	15	3.0 (3)
201-223; 936-958; 1671-1693	PGGTGPGGSGPGGYGPGGSGPGG	910.89 (+2)	15	3.0 (3)
168-192; 903-927; 1638-1662	GSGPGGYGPGGSGPGGSGPGGYGPG	973.88 (+2)	15	3.0 (3)
168-193; 903-928; 1638-1663	GSGPGGYGPGGSGPGGSGPGGYGPGG	1002.37 (+2)	15	3.0 (3)
168-198; 903-933; 1638-1668	GSGPGGYGPGGSGPGGSGPGGYGPGGSGPGG	786.98 (+3)	15	3.0 (3)
169-188; 904-923; 1639-1658	SGPGGYGPGGSGPGGSGPGG	758.29 (+2)	15	3.0 (3)
173-192; 908-927; 1643-1662	GYGPGGSGPGGSGPGGYGPG	796.30 (+2)	15	3.0 (3)
173-198; 908-933; 1643-1668	GYGPGGSGPGGSGPGGYGPGGSGPGG	1002.36 (+2)	15	3.0 (3)
174-198; 909-933; 1644-1668	YGPGGSGPGGSGPGGYGPGGSGPGG	973.87 (+2)	15	3.0 (3)
175-189; 215-229; 910-924; 950-964; 1645-1659; 1685-1699	GPGGSGPGGSGPGGY	360.31 (+3)	30	6.0 (6)
175-204; 215-244; 910-939; 950-979; 1645-1674; 1685-1714	GPGGSGPGGSGPGGYGPGGSGPGGFGPGGS	1181.50 (+2)	30	6.0 (6)
175-209; 215-249; 910-944; 950-984; 1645-1679; 1685-1719	GPGGSGPGGSGPGGYGPGGSGPGGFGPGGSGPGGY	1050.45 (+3)	30	6.0 (6)
175-199; 215-239; 910-934; 950-974; 1645-1669; 1685-1709	GPGGSGPGGSGPGGYGPGGSGPGGF	644.28 (+3)	30	6.0 (6)
178-198; 218-238; 913-930; 953-973; 1648-1668; 1688-1708	GSGPGGSGPGGYGPGGSGPGG	786.81 (+2)	30	6.0 (6)

135-154; 155-174; 180-199; 548-567; 578-597; 598-617; 628-647; 870-889; 890-909; 915-934; 1283-1302; 1313-1332; 1333-1352; 1363-1382; 1605-1624; 1625-1644; 1650-1669; 2018-2037; 2048-2067; 2068-2087; 2098-2117	GPGGSGPGGYGPGGSGPGGY	796.39 (+2)	105	21.0 (21)
249-263; 984-998; 1719-1733	YGPGGSGPGGAGPGG	1144.50 (+1)	15	3.0 (3)
250-269; 985-1004; 1720-1939	GPGGSGPGGAGPGGVGPGGF	709.83 (+2)	15	3.0 (3)
259-268; 381-390; 804-813; 994-1003; 1116-1125; 1539-1548; 1729-1738; 1851-1860; 2254-2283	AGPGGVGPGG	725.32 (+1)	45	9.0 (9)
269-296; 1004-1031; 1739-1766	FGPGGAGPGGAGPGGAGPGGAGPGGAGP	1008.41 (+2)	15	3.0 (3)
288-347; 1023-1082; 1758-1817	GAGPGGAGPGGAGPGGAGPGGAGPGGAGPGGAGPGGAGGAGGAGGAGGSGGAGGSGGTTI	1023.95 (+4)	15	3.0 (3)
310-339; 1045-1074; 1908-1937	GPGGAGPGGAGPGGAGGAGGAGGAGGSGGA	686.61 (+3)	15	3.0 (3)
337-364; 1072-1099; 1807-1834	GGAGGSGGTTIIEDLDITIDGADGPITI	916.05 (+3)	15	3.0 (3)
343-351; 1078-1086; 1813-1821	GGTTIIEDL	918.33 (+1)	15	3.0 (3)
352-368; 775-791; 1087-1103; 1510-1526; 1822-1838; 2245-2261	DITIDGADGPITISEEL	830.42 (+2)	30	6.0 (6)
355-363; 778-786; 1090-1098; 1513-1521; 1825-1833; 2248-2256	IDGADGPIT	858.43 (+1)	30	6.0 (6)
369-396; 792-819; 1104-1131; 1527-1554; 1839-1866; 2262-2289	TISGAGGSGPGGAGPGGVGPGGSGPGGL	1046.54 (+2)	30	6.0 (6)
369-425; 792-848; 1104-1160; 1527-1583; 1839-1895; 2262-2318	TISGAGGSGPGGAGPGGVGPGGSGPGGLGPGGSGPGGVGPGGSGPGGVGPGGAGGPY	1059.75 (+4)	30	6.0 (6)
382-399; 804-822; 1117-1134; 1540-1557; 1872-1869; 2275-2292	GPGGVGPGGSGPGGLGPG	660.28 (+2)	30	6.0 (6)
385-399; 807-822; 1120-1134; 1543-1557; 1875-1869; 2278-2292	GVGPGGSGPGGLGPG	1108.34 (+1)	30	6.0 (6)
390-399; 813-822; 1125-1134; 1548-1557; 1860-1869; 2283-2292	GSGPGGLGPG	741.30 (+1)	30	6.0 (6)
400-419; 1135-1154; 1870-1889	GSGPGGVGPGGSGPGGVGPG	732.29 (+2)	15	3.0 (3)
399-428; 1134-1163; 1869-1898	GGSGPGGVGPGGSGPGGVGPGGAGGPYGPG	771.96 (+3)	15	3.0 (3)

415-433; 1150-1168; 1885-1903	GVGPGGAGGPYGPGGSGPG	727.83 (+2)	15	3.0 (3)
415-436; 1150-1171; 1885-1906	GVGPGGAGGPYGPGGSGPGGAG	820.35 (+2)	15	3.0 (3)
415-437; 1150-1172; 1886-1907	GVGPGGAGGPYGPGGSGPGGAGG	848.85 (+2)	15	3.0 (3)
415-442; 1150-1177; 1886-1912	GVGPGGAGGPYGPGGSGPGGAGGAGGPG	1018.38 (+2)	15	3.0 (3)
415-443; 1150-1178; 1886-1913	GVGPGGAGGPYGPGGSGPGGAGGAGGPGG	1046.92 (+2)	15	3.0 (3)
416-436; 1151-1171; 1886-1906	VGPGGAGGPYGPGGSGPGGAG	791.84 (+2)	15	3.0 (3)
416-443; 1151-1178; 1886-1913	VGPGGAGGPYGPGGSGPGGAGGAGGPGG	679.29 (+3)	15	3.0 (3)
420-429; 461-470; 475-484; 493-502; 517-526; 537-546; 1155-1164; 1196-1205; 1210-1219; 1228-1237; 1252-1261; 1272-1281; 1890-1899; 1931-1940; 1945-1954; 1963-1972; 1987-1996; 2007-2016	GAGGPYGPGG	789.34 (+1)	90	18.0 (18)
420-443; 1155-1178; 1890-1913	GAGGPYGPGGSGPGGAGGAGGPGG	863.35 (+2)	15	3.0 (3)
426-445; 1161-1180; 1896-1915	GPGGSGPGGAGGAGGPGGAY (*) nitrotyrosine	751.88 (+2)	15	3.0 (3)
426-451; 1161-1186; 1896-1921	GPGGSGPGGAGGAGGPGGAYGPGGSY	758.38 (+3)	15	3.0 (3)
446-451; 1181-1186; 1916-1921	GPGGSY	817.23 (+1)	15	3.0 (3)
446-466; 1181-1201; 1916-1936	GPGGSYGPGGSGGPGGAGGPY	831.94 (+2)	15	3.0 (3)
446-480; 1181-1215; 1916-1950	GPGGSYGPGGSGGPGGAGGPYGPGGEGPGGAGGPY	920.07 (+3)	15	3.0 (3)
446-454; 1181-1189; 1916-1924	GPGGSYGPG	748.25 (+1)	15	3.0 (3)
446-465; 1181-1201; 1916-1936	GPGGSYGPGGSGGPGGAGGPY	831.93 (+2)	15	3.0 (3)
450-474; 1185-1209; 1920-1944	SYGPGGSGGPGGAGGPYGPGGEGPG	1001.89 (+2)	15	3.0 (3)
451-460; 1186-1195; 1921-1930	YGPGGSGGPG	805.31 (+1)	15	3.0 (3)
451-483; 1186-1218; 1921-1953	YGPGGSGGPGGAGGPYGPGGEGPGGAGGPYGPG	877.03 (+3)	15	3.0 (3)
452-466; 1187-1201; 1922-1936	GPGGSGGPGGAGGPY	580.75 (+2)	15	3.0 (3)
452-480; 1187-1215; 1922-1950	GPGGSGGPGGAGGPYGPGGEGPGGAGGPY	757.66 (+3)	15	3.0 (3)
452-489; 1187-1124; 1923-1959	GPGGSGGPGGAGGPYGPGGEGPGGAGGPYGPGGAGGPY	791.10 (+4)	15	3.0 (3)

452-466; 1187-1201; 1922-1936	GPGGSGGPGGAGGPY	572.75 (+2)	15	3.0 (3)
452-480; 1187-1215; 1922-1950	GPGGSGGPGGAGGPYGPGGEGPGGAGGPY	770.35 (+3)	15	3.0 (3)
452-489; 1187-1124; 1923-1959	GPGGSGGPGGAGGPYGPGGEGPGGAGGPYGPGGAGGPY	990.09 (+3)	15	3.0 (3)
461-474; 1196-1209; 1931-1944	GAGGPYGPGGEGPG	1129.45 (+1)	15	3.0 (3)
461-483; 1196-1218; 1931-1953	GAGGPYGPGGEGPGGAGGPYGPG	921.92 (+2)	15	3.0 (3)
461-497; 1196-1232; 1931-1967	GAGGPYGPGGEGPGGAGGPYGPGGAGGPYGPGGAGGP	921.86 (+2)	15	3.0 (3)
467-480; 529-542; 1202-1215; 1264-1277; 1937-1950; 1999-2012	GPGGEGPGGAGGPY	965.73 (+3)	30	6.0 (6)
467-489; 1202-1224; 1937-1959	GPGGEGPGGAGGPYGPGGAGGPY	929.90 (+2)	15	3.0 (3)
467-498; 1202-1233; 1937-1968	GPGGEGPGGAGGPYGPGGAGGPYGPGGAGGPY	714.78 (+4)	15	3.0 (3)
467-489; 1202-1224; 1937-1959	GPGGEGPGGAGGPYGPGGAGGPY	930.46 (+2)	15	3.0 (3)
475-497; 1210-1232; 1945-1967	GAGGPYGPGGAGGPYGPGGAGGP	892.87 (+2)	15	3.0 (3)
417-425; 458-466; 472-480; 481-489; 490-498; 499-507; 514-522; 534-542; 1152-1160; 1193-1201; 1207-1215; 1216-1224; 1225-1233; 1234-1242; 1249-1257; 1269-1277; 1887-1895; 1928-1939; 1942-1950; 1951-1959; 1960-1968; 1969-1977; 1984-1992; 2004-2012	GPGGAGGPY	366.66 (+2)	120	24.0 (24)
472-507; 1207-1242; 1942-1977	GPGGAGGPYGPGGAGGPY (*) dihydroxyproline / phosphotyrosine	779.39 (+2)	15	3.0 (3)
472-507; 1207-1242; 1942-1977	GPGGAGGPYGPGGAGGPY (*) phosphotyrosine	763.41 (+2)	15	3.0 (3)
472-516; 1207-1251; 1942-1986	GPGGAGGPYGPGGAGGPYGPGGEGGPY	1108.98 (+2)	15	3.0 (3)
463-516; 1198-1251; 1933-1986	GPGGAGGPYGPGGEGGPY	752.40 (+2)	15	3.0 (3)
417-425; 458-466; 472-480; 481-489; 490-498; 499-507; 514-522; 534-542; 1152-1160; 1193-1201; 1207-1215; 1216-1224; 1225-1233; 1234-1242; 1249-1257; 1269-1277; 1887-1895; 1928-1936; 1942-1950; 1951-1959; 1960-1968; 1969-1977; 1984-1992; 2004-2012	GPGGAGGPY	732.38 (+1)	120	24.0 (24)
499-513; 1234-1248; 1969-1983	GPGGAGGPYGPGGPD	651.79 (+2)	15	3.0 (3)

499-522; 1234-1257; 1969-1992	GPGGAGGPYGPGGPDGPGGAGGPY	1017.01 (+2)	15	3.0 (3)
508-522; 1243-1257; 1978-1992	GPGGPDGPGGAGGPY	631.30 (+2)	15	3.0 (3)
508-528; 1243-1263; 1978-1998	GPGGPDGPGGAGGPYGPGGPY	886.98 (+2)	15	3.0 (3)
513-536; 1248-1271; 1983-2006	YGPGGAGGPYGPGGPYGPGGEGPG	1016.40 (+2)	15	3.0 (3)
514-528; 1249-1263; 1984-1998	GPGGAGGPYGPGGPY (*) hydroxyproline	638.80 (+2)	15	3.0 (3)
514-542; 1249-1277; 1984-2012	GPGGAGGPYGPGGPYGPGGEGPGGAGGPY	791.10 (+3)	15	3.0 (3)
502-512; 517-527; 1237-1247; 1252-1262; 1972-1982; 1987-1997	GAGGPYGPGGP	886.35 (+1)	30	6.0 (6)
517-536; 1252-1271; 1987-2006	GAGGPYGPGGPYGPGGEGPG	829.37 (+2)	15	3.0 (3)
517-546; 1252-1281; 1987-2016	GAGGPYGPGGPYGPGGEGPGGAGGPYGPGG	809.98 (+3)	15	3.0 (3)
519-536; 1254-1271; 1988-2006	GGPYGPGGPYGPGGEGPG	765.32 (+2)	15	3.0 (3)
519-546; 1254-1281; 1988-2016	GGPYGPGGPYGPGGEGPGGAGGPYGPGG	767.29 (+3)	15	3.0 (3)
522-546; 1257-1281; 1992-2016	YGPGGPYGPGGEGPGGAGGPYGPGG	1044.89 (+2)	15	3.0 (3)
523-542; 1258-1277; 1993-2012	GPGGPYGPGGEGPGGAGGPY	837.36 (+2)	15	3.0 (3)
529-557; 1264-1292; 1999-2027	GPGGEGPGGAGGPYGPGGVGPGGSGPGGY	1142.00 (+2)	15	3.0 (3)
529-567; 1264-1302; 1999-2037	GPGGEGPGGAGGPYGPGGVGPGGSGPGGYGPGGSGPGGY	1018.78 (+3)	15	3.0 (3)
543-567; 1278-1302; 2013-2037	GPGGVGPGGSGPGGYGPGGSGPGGY	667.62 (+3)	15	3.0 (3)
543-577; 1278-1312; 2013-2047	GPGGVGPGGSGPGGYGPGGSGPGGYGPGGAGPGGY	906.39 (+3)	15	3.0 (3)
115-134; 558-577; 850-869; 12936-1312; 1585-1604; 2028-2047	GPGGSGPGGYGPGGAGPGGY	825.39 (+2)	30	6.0 (6)
115-144; 558-587; 850-879; 12936-1322; 1585-1614; 2028-2057	GPGGSGPGGYGPGGAGPGGYGPGGSGPGGY	868.22 (+3)	30	6.0 (6)
608-627; 1343-1362; 2078-2097	GPGGSGPGGYGSGGAGPGGY	741.96 (+2)	15	3.0 (3)
608-637; 1343-1372; 2078-2107	GPGGSGPGGYGSGGAGPGGYGPGGSGPGGY	811.31 (+3)	15	3.0 (3)
618-627; 1353-1362; 2088-2097	GSGGAGPGGY	913.51 (+1)	15	3.0 (3)
618-637; 1353-1372; 2088-2107	GSGGAGPGGYGPGGSGPGGY	804.92 (+2)	15	3.0 (3)

628-667; 1363-1402; 2098-2137	GPGGSGPGGYGPGGSGPGGYGPGGTGPGGTGPGGSGPGGY	1055.78 (+3)	15	3.0 (3)
638-687; 1373-1422; 2108-2157	GPGGSGPGGYGPGGTGPGGTGPGGSGPGGYGPGGSGPGRYGPGGSGPGGY	965.44 (+4)	15	3.0 (3)
661-686; 1396-1421; 2131-2156	GSGPGGYGPGGSGPGRYGPGGSGPGG	964.36 (+2)	15	3.0 (3)
668-687; 1403-1422; 2138-2157	GPGGSGPGRYGPGGSGPGGY	796.40 (+2)	15	3.0 (3)
668-697; 1403-1432; 2138-2167	GPGGSGPGRYGPGGSGPGGYGPSGSGPGGY	608.61 (+4)	15	3.0 (3)
688-697; 698-707; 1423-1432; 1433-1442; 2158-2167; 2168-2177	GPSGSGPGGY	449.69 (+2)	30	6.0 (6)
698-717; 1433-1452; 2168-2187	GPSGSGPGGYGPSGSGPGGY	549.25 (+3)	15	3.0 (3)
718-774; 1453-1509; 2188-2244	GPGGSGAGGTGPGGAGGAGGAGGSGGAGGSGGAGGSGGAGGSGGVGGSGGTTITEDL	1042.70 (+4)	15	3.0 (3)
823-837; 1558-1572; 2293-2307	GPGGSGSGGVGPGGY	621.76 (+2)	15	3.0 (3)
823-845; 1558-1580; 2293-2315	GPGGSGSGGVGPGGYGPGGSGGF	889.89 (+2)	15	3.0 (3)
823-846; 1558-1581; 2293-2316	GPGGSGSGGVGPGGYGPGGSGGFY	971.42 (+2)	15	3.0 (3)
	**Non-repetitive C-terminal domain**			
2316-2325	YGPGGSEGPY	482.74 (+2)	8	1.6 (2)
2317-2325	GPGGSEGPY	533.26 (+2)	6	1.2 (1)
2317-2331	GPGGSEGPYGPSGTY	691.79 (+2)	9	1.8 (2)
2326-2337	GPSGTYGSGGGY	642.79 (+2)	5	1.0 (1)
2332-2346	GSGGGYGPGSAGGPN	620.76 (+2)	5	1.0 (1)
2332-2355	GSGGGYGPGSAGGPNGPGSPGGAY	635.62 (+3)	6	1.2 (1)
2347-2364	GPGSPGGAYGPGSPGGAY	712.80 (+2)	7	1.4 (1)
2356-2391	GPGSPGGAYYPSSRVPDMVNGIMSAMQGSGFNYQMF	1024.17 (+4)	5	1.0 (1)
2365-2386	YPSSRVPDMVNGIMSAMQGSGF	794.01 (+3)	6	1.2 (1)
2365-2388	YPSSRVPDMVNGIMSAMQGSGFNY	860.40 (+3)	8	1.6 (2)
2387-2395	NYQMFGNML	617.84 (+2)	9	1.8 (2)
2389-2395	QMFGNML	421.77 (+2)	9	1.8 (2)

2396-2413	SQYSSGSGTCNPNNVNVL	610.71 (+3)	5	1.0 (1)
2396-2417	SQYSSGSGTCNPNNVNVLMDAL	1113.99 (+2)	5	1.0 (1)
2399-2417	SSGSGTCNPNNVNVLMDAL	674.07 (+3)	6	1.2 (1)
2399-2418	SSGSGTCNPNNVNVLMDALL	1032.48 (+2)	8	1.6 (2)
2419-2441	HCLSNHGSSSFAPSPTPAAMSAY	858.15 (+3)	6	1.2 (1)
2422-2429	SNHGSSSF	412.72 (+2)	7	1.4 (1)
2442-2449	SNSVGRMF	527.27 (+2)	8	1.6 (2)
2430-2451	APSPTPAAMSAYSNSVGRMFAY	794.84 (+3)	5	1.0 (1)

Supplementary figures requested by Sientific Reports demonstrating the full-length gel and blots results.

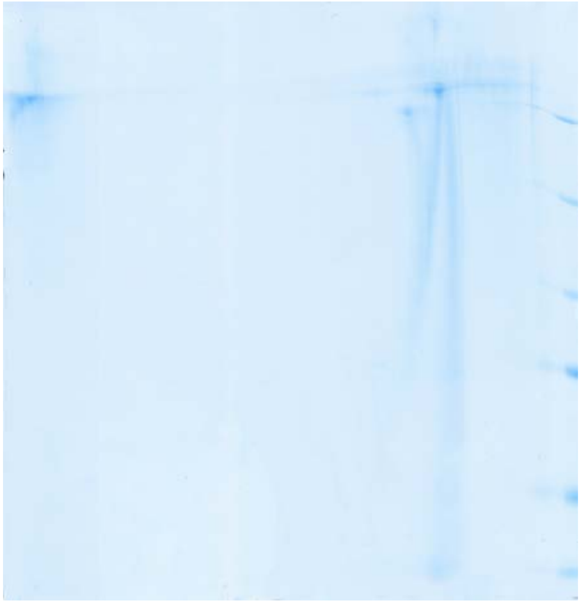

Figure S7. Representative 2-DE profile of the flagelliform silk stained with *Coomassie Colloidal Blue*.

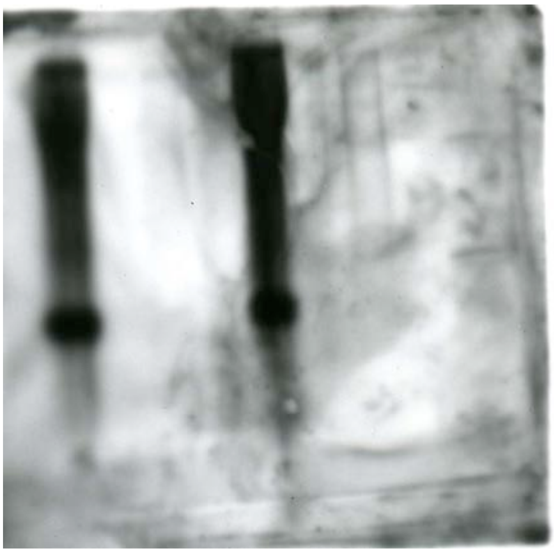

Figure S8. Western blotting showing phosphotyrosine immunoreactivity.

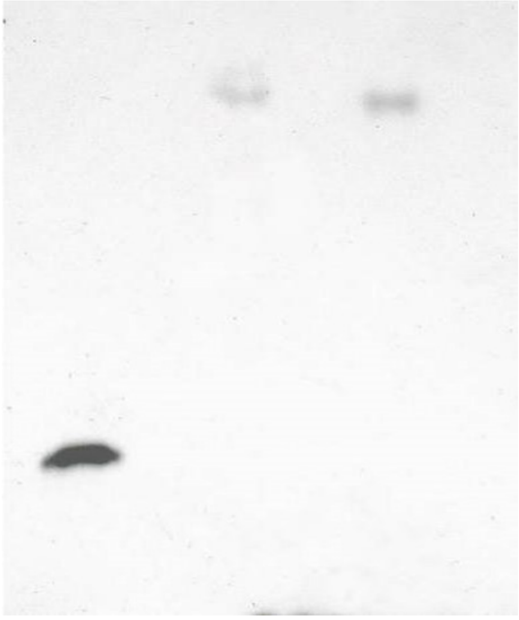

Figure S9. Western blotting showing nitrotyrosine immunoreactivity.
